# Flavonoid Preparations from *Taraxacum officinale* L. Fruits—A Phytochemical, Antioxidant and Hemostasis Studies

**DOI:** 10.3390/molecules25225402

**Published:** 2020-11-18

**Authors:** Bernadetta Lis, Dariusz Jedrejek, Joanna Rywaniak, Agata Soluch, Anna Stochmal, Beata Olas

**Affiliations:** 1 Department of General Biochemistry, Faculty of Biology and Environmental Protection, University of Lodz, 90-236 Lodz, Poland; bernadetta.lis@biol.uni.lodz.pl; 2 Department of Biochemistry and Crop Quality, Institute of Soil Science and Plant Cultivation, State Research Institute, 24-100 Pulawy, Poland; djedrejek@iung.pulawy.pl (D.J.); asoluch@iung.pulawy.pl (A.S.); asf@iung.pulawy.pl (A.S.); 3 Department of Immunology and Infectious Biology, Institute of Microbiology, Biotechnology and Immunology, Faculty of Biology and Environmental Protection, University of Lodz, 90-237 Lodz, Poland; joanna.rywaniak@biol.uni.lodz.pl

**Keywords:** dandelion fruits, HR-QTOF-MS, flavones, biflavones, flavonolignans, antiplatelet activity, adhesion, blood platelets, oxidative stress

## Abstract

Dandelion (*Taraxacum officinale* L.) roots, leaves, and flowers have a long history of use in traditional medicine. Compared to the above organs, dandelion fruits are the least known and used. Hence, the present paper was aimed at the phytochemical analysis of *T. officinale* fruit extract and estimating its antiradical, antiplatelet, and antioxidant properties related to hemostasis. Methanolic extract of fruits (E1), enriched with polyphenols (188 mg gallic acid equivalents (GAE)/g), was successfully separated into cinnamic acids (E2; 448 mg GAE/g) and flavonoids (E3; 377 mg GAE/g) extracts. Flavonoid extract was further divided into four fractions characterized by individual content: A (luteolin fraction; 880 mg GAE/g), B (philonotisflavone fraction; 516 mg GAE/g), C (flavonolignans fraction; 384 mg GAE/g), and D (flavone aglycones fraction; 632 mg GAE/g). High DPPH radical scavenging activity was evaluated for fractions A and B (A > B > Trolox), medium for extracts (Trolox > E3 > E2 > E1), and low for fractions C and D. No simple correlation between polyphenol content and antiradical activity was observed, indicating a significant influence of qualitative factor, including higher anti-oxidative effect of flavonoids with B-ring catechol system compared to hydroxycinnamic acids. No cytotoxic effect on platelets was observed for any dandelion preparation tested. In experiments on plasma and platelets, using several different parameters (lipid peroxidation, protein carbonylation, oxidation of thiols, and platelet adhesion), the highest antioxidant and antiplatelet potential was demonstrated by three fruit preparations–hydroxycinnamic acids extract (E2), flavonoid extract (E3), and luteolin fraction (A). The results of this paper provide new information on dandelion metabolites, as well as their biological potential and possible use concerning cardiovascular diseases.

## 1. Introduction

The occurrence of some civilization diseases, such as atherosclerosis, diabetes, heart attack, and some cancers, has been associated with elevated levels of oxidative stress and dysfunctional hemostasis. Hemostasis is defined as a sequence of reactions that maintain the fluidity of circulating blood, while also preventing its outflow in the event of a break in the vessel [1,2]. However, the structure and functioning of the biomolecules involved in hemostasis can be impaired by the effects of reactive oxygen species (ROS) [3]. ROS concentrations can be increased in response to biotic factors such as enzymatic reactions, oxidation of respiratory proteins or oxidation of xenobiotics, as well as abiotic factors such as ionizing radiation, environmental pollution, toxins, drugs, and an unhealthy diet. In contrast, many studies indicate that the consumption of products rich in natural antioxidants, i.e., certain foods and nutraceuticals, has a positive effect on maintaining the oxidant-antioxidant balance in the body [4,5]. Many such substances with antiradical and antioxidant properties have been identified so far, and some, such as ascorbic acid, masoprocol, pramipexole, allopurinol, vitamin A or vitamin E [6], are currently used as components of drugs or dietary supplements. Nevertheless, new compounds with such properties are constantly being sought.

Medical plants and their extracts are also particularly well-exploited sources of natural antioxidants. Of these, the last few decades have seen a growth in interest in the dandelion, particularly *T. officinale*, as a source of bioactive compounds bestowing anti-inflammatory, antioxidative, choleretic, diuretic, hepatoprotective, and immunostimulatory effects [7]. In particular, the antioxidant and anti-inflammatory activities of dandelion extracts and preparations have been mainly attributed to their polyphenolic components [8,9]. The most abundant phenolic compounds found in either *Taraxacum* root or aerial parts are hydroxycinnamic acid derivatives (HCAs), such as chicoric (dicaffeoyltartaric), caftaric (monocaffeoyltartaric), and chlorogenic acids [10,11]. Additionally, many flavonoids, such as free aglycones and flavonoid glycosides, have been identified in both flower and leaf extracts, with the most commonly-reported examples being the flavones luteolin, chrysoeriol and apigenin, and various flavonols, such as quercetin, and their *O*-glycosides glucoside and rutinoside [12,13].

Dandelion fruits are straw- to brown-colored achenes and lanceolate in shape. Moreover, the fruits are 3–5 mm long and equipped with characteristic silky pappi to allow wind dispersion. However, very few, if any, studies on *T. officinale* have examined the phytochemical composition and biological properties of its fruit, most likely because they are not regarded as herbal material, unlike the more widely-used roots, leaves and flowers. In the only study, the ethanol extract of *T. officinale* fruits showed free radical scavenging activity and protective effect against oxidation of rat brain cells (neuroprotective activity). The positive action was finally correlated with an elevated phenolic content in the plant extract, however, more detailed information on its chemical composition was lacking [14].

The current study was aimed at the phytochemical analysis of *T. officinale* fruit extract and estimating its antiradical, antiplatelet, and antioxidant properties related to hemostasis. Efforts were also made to estimate the contribution of individual polyphenols on the reported biological activity of dandelion fruits.

## 2. Results

### 2.1. Phytochemical Characteristics of Plant Extracts and Fractions

**Figure 1:**
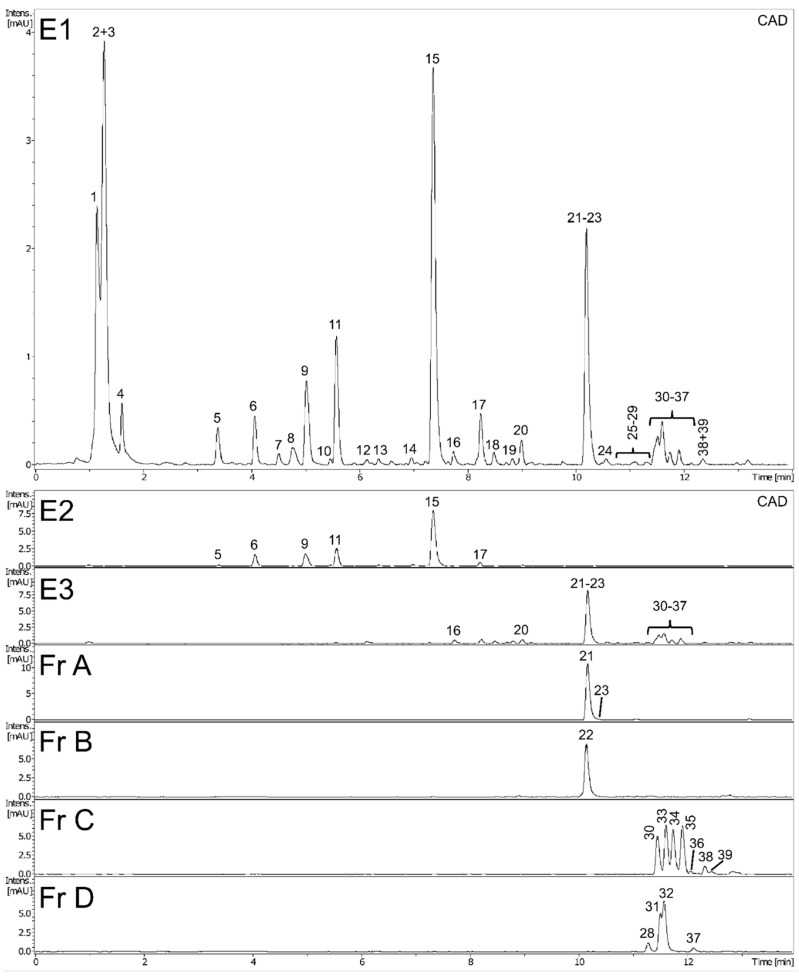
Phytochemical analysis of extracts (E1–E3) and flavonoid fractions (A–D) of dandelion fruits.

Preliminary UHPLC-PDA-CAD-ESI-QTOF-MS/MS analysis revealed the presence of numerous compounds in methanol extract (E1) of dandelion fruits. The peaks were tentatively identified and classified based on MS and UV spectra and using the SIRIUS 4 tool [15] and the literature; while most appeared to be phenolic derivatives, several unidentified nitrogen-containing metabolites were also detected (Figure 1 and Table 1, peaks were assigned by retention time). Next, the full identity of 14 metabolites was confirmed by comparison with possessed authentic standard compounds (Table 1); these included four caffeic acid esters (5-*O*-caffeoylquinic, 3,5-di-caffeoylquinic, caffeic and L-chicoric acid), nine flavonoids (7-, 4’-, and 3’-*O*-glucosides of luteolin, apigenin, apometzgerin, chrysoeriol, luteolin, philonotisflavone, and tricin) and a sesquiterpene lactone (taraxinic acid-1’-*O*-glucoside). Structures of known described compounds are shown in Figure S2. Consequently, the dominant phenolic constituents in extract E1 of dandelion fruits were found to be hydroxycinnamic acid derivatives (HCAs) and flavone derivatives, which are known constituents of leaves and flowers, the most widely-studied dandelion aerial organs. Besides, several metabolites not yet reported in *T. officinale*, such as biflavones and some flavonolignans, were also detected (Table 1).

Subsequent multistep fractionation of methanolic extract of dandelion fruits (E1) allowed its successful separation into phenolic acid extract (E2) and flavonoid extract (E3), then extract E3 was further divided into four flavonoid fractions A–D (Figure S1). The phytochemical profiles of all dandelion preparations were examined in detail using UHPLC-PDA-CAD-ESI-QTOF-MS/MS analyses. As can be seen in Figure 1, showing CAD chromatograms of dandelion extracts and fractions (peak numbering according to Table 1), each preparation has an individual composition. Consequently, prepared flavonoid fractions A–D can easily be described according to their composition: A (luteolin fraction), B (philonotisflavone fraction), C (flavonolignan fraction), and D (flavone aglycone fraction).

The contents of hydroxycinnamic acids and flavonoids in dandelion fruit extracts (E1–E3) and fractions (A–D) were evaluated by UHPLC-PDA and expressed as mg L-chicoric acid or luteolin equivalents per gram DW of extract/fraction (Table 2). Caffeic acid derivatives, mainly L-chicoric acid, were the dominant polyphenols in the extract E1 (about 150 mg/g DW) and E2 (about 530 mg/g DW); in turn, the HCAs were a minor component of extract E3 (about 30 mg/g DW) and were completely absent from fractions A–D. The major phenolic compounds in the flavonoid preparations were flavone derivatives (E3 and Fr A–D, in the range between 280–995 mg/g DW); these were sometimes the only polyphenols present.

Additionally, the total phenolic content (TPC) in extracts and fractions of dandelion fruits was estimated by the Folin-Ciocalteu assay (Table 2). In general, the values obtained by TPC were comparable with those of the UHPLC assay, as indicated by the color-coded results given in Table 2. The highest TPC values were found in fractions A and D (880 and 632 mg GAE/g, respectively), and the lowest in extracts E1 and E3 (188 and 377 mg GAE/g, respectively).

### 2.2. DPPH Free Radical Scavenging Activity

The DPPH radical scavenging activity of dandelion fruits phenolic preparations (E1–E3, Fr A–D) was expressed as Trolox Equivalents (TE) and IC_50_ values (Table 3). The estimated TE values varied from 0.05 to 2.01 of Trolox activity, with 1.00 indicating equivalence to Trolox. Fraction A (luteolin) and B (philonotisflavone) demonstrated the highest scavenging activity (2.01 and 1.09 TE, respectively), followed by the three extracts E1–E3 (0.26–0.55 TE), and the fractions D and C (0.05 and 0.06 TE, respectively). The antioxidant capacity of the tested samples (dandelion preparations and Trolox) proceeded in the following order: Fr A (luteolin) > Fr B (philonotisflavone) > Trolox > E3 (flavonoid extract) > E2 (phenolic acid extract) > E1 (total extract) > Fr C (flavonolignans) > Fr D (flavone aglycones).

### 2.3. Biomarkers of Oxidative Stress in Plasma and Blood Platelets

**Figure 2:**
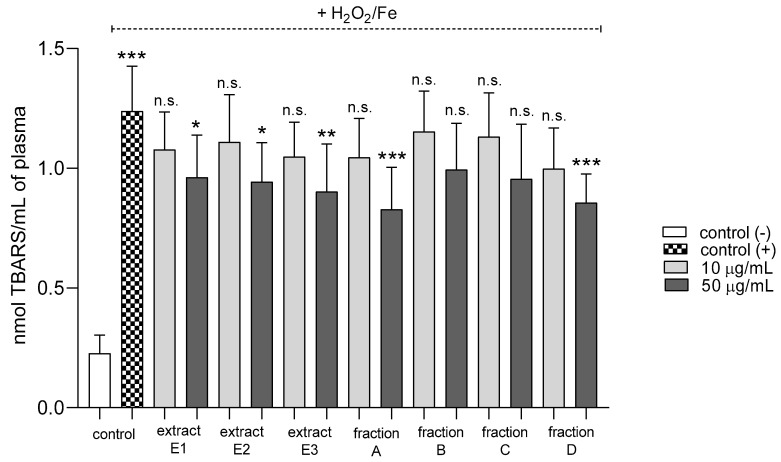
Effects of extracts (E1–E3) and flavonoid fractions (A–D) of dandelion fruits (concentrations 10 and 50 µg/mL) on lipid peroxidation in plasma treated with H_2_O_2_/Fe. The data are presented as means ± SD (*n* = 10). Control negative refers to plasma not treated with H_2_O_2_/Fe, and control positive to plasma treated with H_2_O_2_/Fe. Kruskal-Wallis test: n.s. *p* > 0.05, * *p* < 0.05, ** *p* < 0.01, *** *p* < 0.001, compared with positive control.

**Figure 3:**
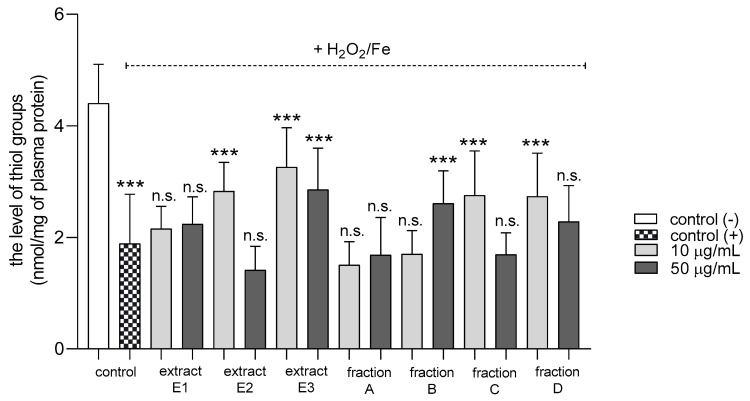
Effects of extracts (E1–E3) and flavonoid fractions (A–D) of dandelion fruits (concentrations 10 and 50 µg/mL) on the level of thiol groups in plasma treated with H_2_O_2_/Fe. The data are presented as means ± SD (*n* = 8). Control negative refers to plasma not treated with H_2_O_2_/Fe, and control positive to plasma treated with H_2_O_2_/Fe. Kruskal-Wallis test: n.s. *p* > 0.05, *** *p* < 0.001, compared with positive control.

Three extracts E1–E3 and two fractions A and D inhibited plasma lipid peroxidation stimulated by H_2_O_2_/Fe at the highest tested concentration (50 µg/mL); however, fractions B and C remained inactive (Figure 2). In addition, extracts E2 and E3 and fractions B, C, and D reduced protein thiol group oxidation in plasma treated with H_2_O_2_/Fe (Figure 3); extract E3 reduced this process at 10 and 50 µg/mL (Figure 3).

**Figure 4:**
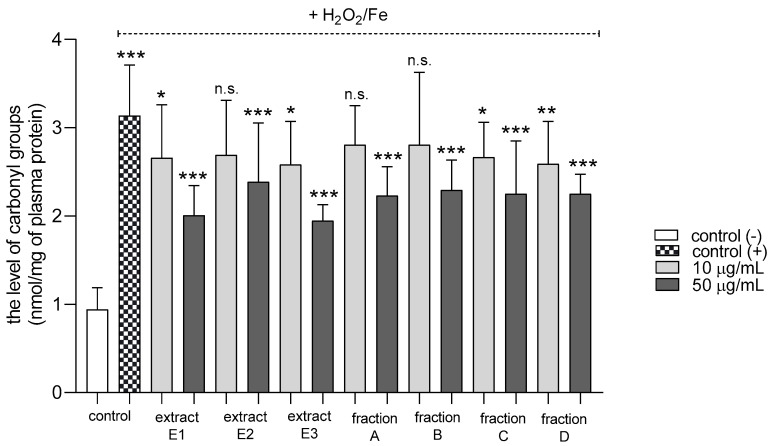
Effects of extracts (E1–E3) and flavonoid fractions (A–D) of dandelion fruits (concentrations 10 and 50 µg/mL) on the levels of carbonyl groups in plasma treated with H_2_O_2_/Fe. The data are presented as means ± SD (*n* = 8). Control negative refers to plasma not treated with H_2_O_2_/Fe, and control positive to plasma treated with H_2_O_2_/Fe. Kruskal-Wallis test: n.s. *p* > 0.05, * *p* < 0.05, ** *p* < 0.01, *** *p* < 0.001, compared with positive control.

All tested dandelion fruit preparations inhibited protein carbonylation in plasma treated with H_2_O_2_/Fe at the highest tested concentration (50 µg/mL) (Figure 4). In addition, two extracts (E1 and E3) and two fractions (C and D) were also active at the lower tested concentration (10 µg/mL).

**Figure 5:**
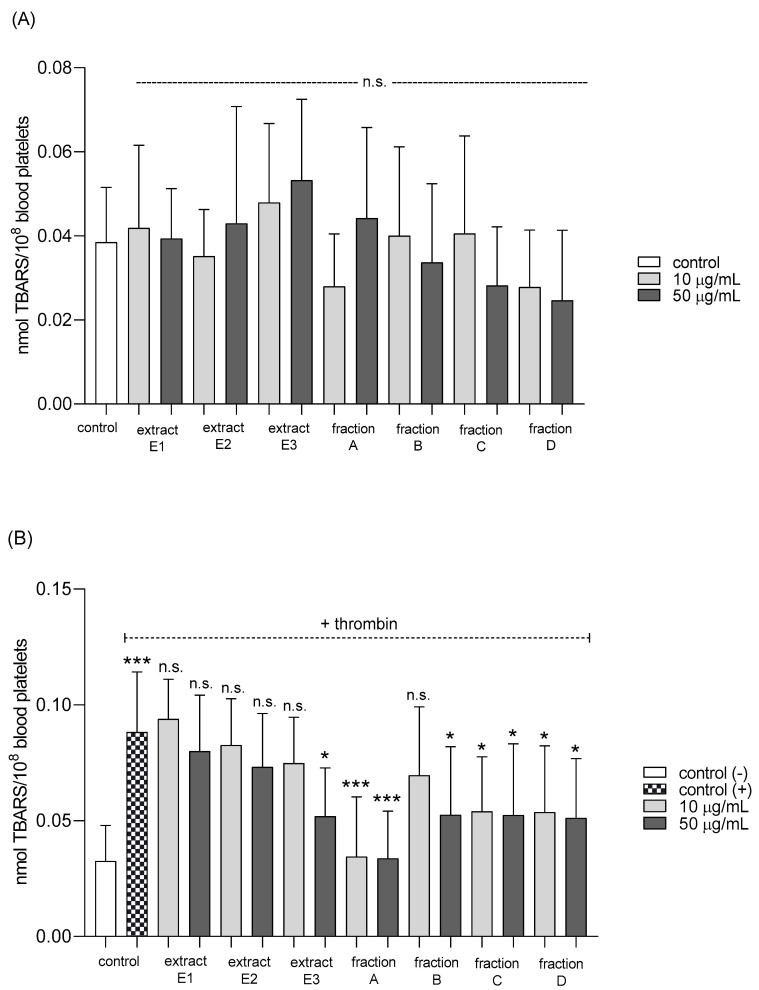

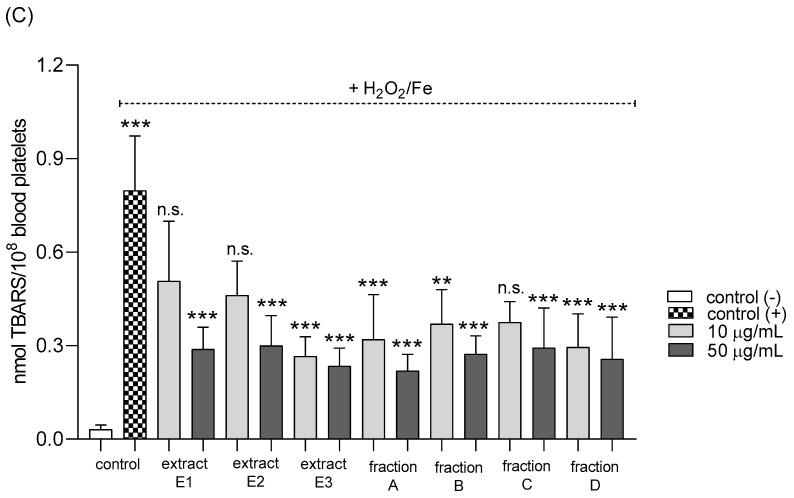
Effects of extracts (E1–E3) and flavonoid fractions (A–D) of dandelion fruits (concentrations 10 and 50 µg/mL) on lipid peroxidation in resting blood platelets (**A**), platelets activated by 5 U/mL thrombin (**B**), and platelets treated with H_2_O_2_/Fe (**C**). The data are presented as means ± SD (*n* = 5). Control negative refers to platelets not treated with thrombin or H_2_O_2_/Fe, and control positive to platelets treated with thrombin or H_2_O_2_/Fe. Kruskal-Wallis test: n.s. *p* > 0.05, * *p* < 0.05, ** *p* < 0.01, *** *p* < 0.001.

**Figure 6:**
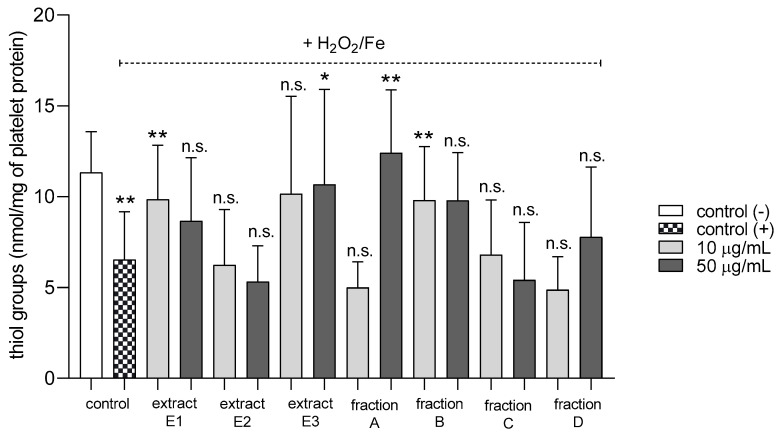
Effects of extracts (E1–E3) and flavonoid fractions (A–D) of dandelion fruits (concentrations 10 and 50 µg/mL) on the protein thiol group content of platelets treated with H_2_O_2_/Fe. The data are presented as means ± SD (*n* = 5). Control negative refers to platelets not treated with H_2_O_2_/Fe, and control positive refers to platelets treated with H_2_O_2_/Fe. Kruskal-Wallis test: n.s. *p* > 0.05, * *p* < 0.05, ** *p* < 0.01, compared with positive control.

**Figure 7:**
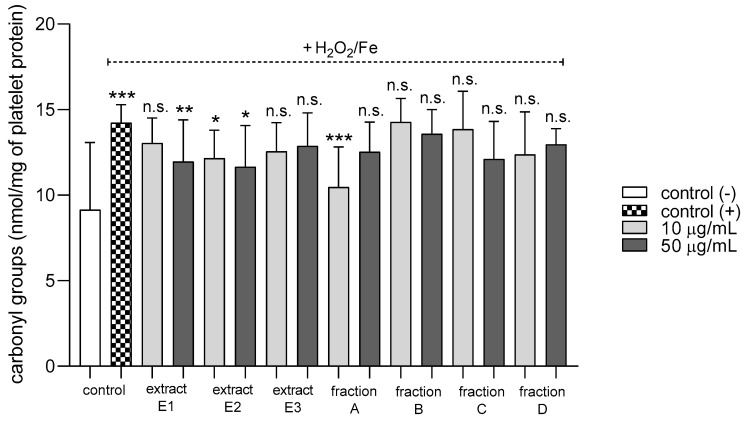
Effects of extracts (E1–E3) and flavonoid fractions (A–D) of dandelion fruits (concentrations 10 and 50 µg/mL) on the level of carbonyl groups in platelet protein treated with H2O2/Fe. The data are presented as means ± SD (*n* = 5). Control negative refers to platelets not treated with H_2_O_2_/Fe, and control positive to platelets treated with H_2_O_2_/Fe. Kruskal-Wallis test: n.s. *p* > 0.05, * *p* < 0.05, ** *p* < 0.01, *** *p* < 0.001, compared with positive control.

None of the tested preparations changed the TBARS level in resting platelets at either 10 or 50 µg/mL (Figure 5A). However, extract E3 and fractions A–D significantly inhibited thrombin-induced platelet lipid peroxidation when administered at 50 µg/mL. For example, 10 and 50 µg/mL luteolin fraction inhibited this process by about 60% compared with positive controls (Figure 5B). In addition, three flavone fractions (A, B, and D) significantly reduced lipid peroxidation induced by H_2_O_2_/Fe when administered at 10 and 50 µg/mL; however, two of the extracts (E1 and E2) and fraction C (10 µg/mL) did not appear to exert any activity (Figure 5C). H_2_O_2_/Fe-induced oxidation of protein thiol functions was ameliorated by preparations E1 and B at 10 µg/mL, and by E3 and A at 50 µg/mL (Figure 6). Moreover, H_2_O_2_/Fe-induced protein carbonylation was inhibited by extracts E1, E2 and fraction A (Figure 7); for example, phenolic acid extract (E2) demonstrated a protective effect at two used doses (10 and 50 µg/mL) (Figure 7).

### 2.4. Hemostatic Parameters of Blood Platelets and Plasma

**Figure 8:**
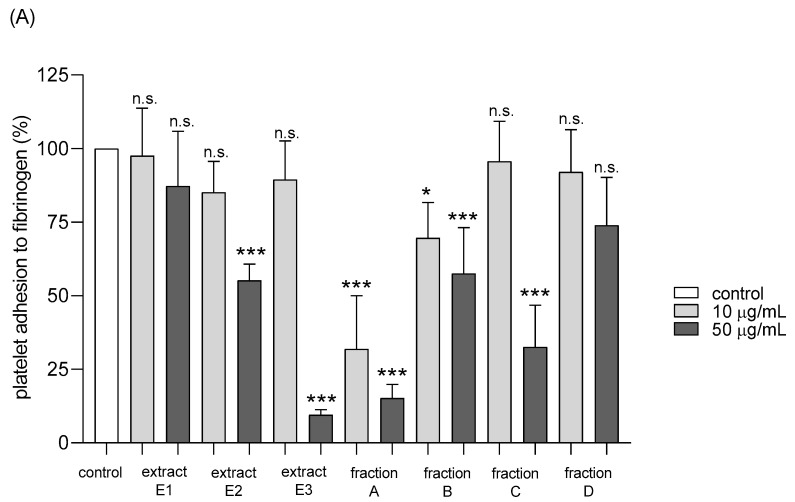

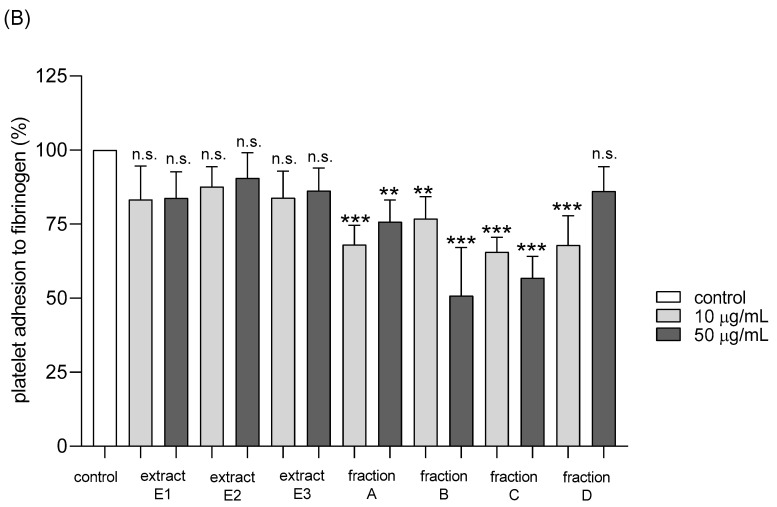
Effects of extracts (E1–E3) and flavonoid fractions (A–D) of dandelion fruits (concentrations 10 and 50 µg/mL) on adhesion to fibrinogen and ADP-activated platelets (**A**), and thrombin-activated platelets (**B**). The data are presented as percentages of the control sample (platelets without plant preparation). Results are given as means ± SD (*n* = 5). Kruskal-Wallis test: n.s. *p* > 0.05, * *p* < 0.05, ** *p* < 0.01, *** *p* < 0.001, compared with control.

Adhesion of thrombin-activated platelets to fibrinogen was significantly inhibited after preincubation with five tested dandelion fruit preparations (E2 and E3, fractions A, B, and C) (Figure 8A). Fraction A was highly active at both concentrations tested—10 and 50 µg/mL, while the others (E2, E3, B, and C) at the higher concentration (Figure 8A). In the case of ADP-activated blood platelets, all four flavonoid fractions demonstrated inhibitory action, fractions A–C at both tested doses (10 and 50 µg/mL), fraction D only at 10 µg/mL (Figure 8B).

**Figure 9:**
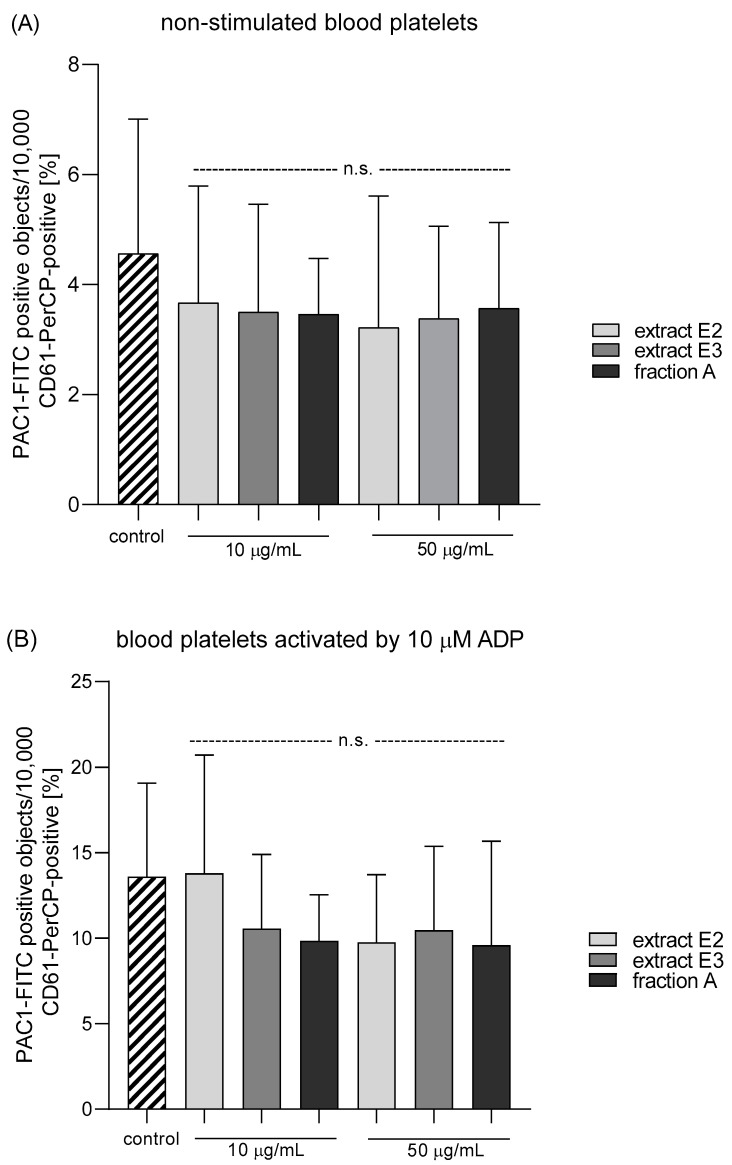

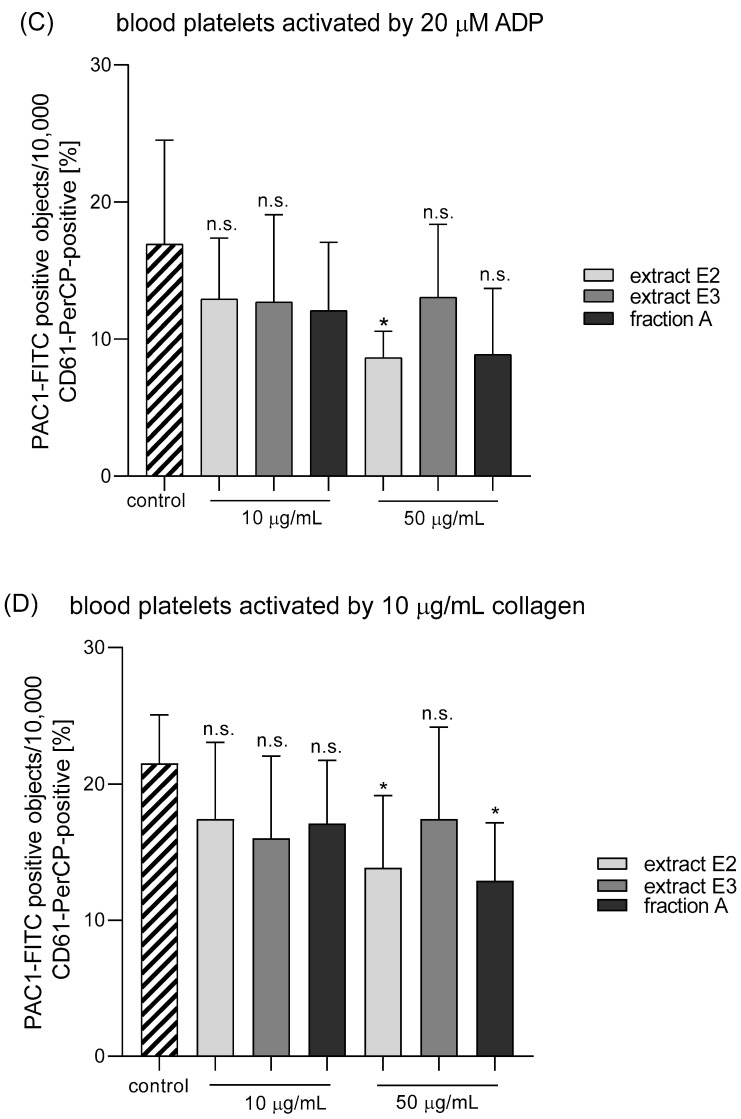
Effects of dandelion fruit preparations (E2 and E3, and fraction A; concentrations 10 and 50 µg/mL) on the expression of the active form of GPIIb/IIIa in resting (**A**) or agonist-stimulated blood platelets: 10 µM ADP (**B**), 20 µM ADP (**C**) and 10 µg/mL collagen (**D**) in whole blood samples. The blood platelets were distinguished based on the expression of CD61. For each sample, 10,000 CD61-positive objects (blood platelets) were acquired. For the assessment of GPIIb/IIIa expression, samples were labeled with fluorescently conjugated monoclonal antibody PAC-1/FITC. Results are expressed as the percentage values of platelets binding PAC-1/FITC. Data represent the means ± SE of six healthy volunteers. n.s. *p* > 0.05; * *p* < 0.05 (vs. control platelets).

**Figure 10:**
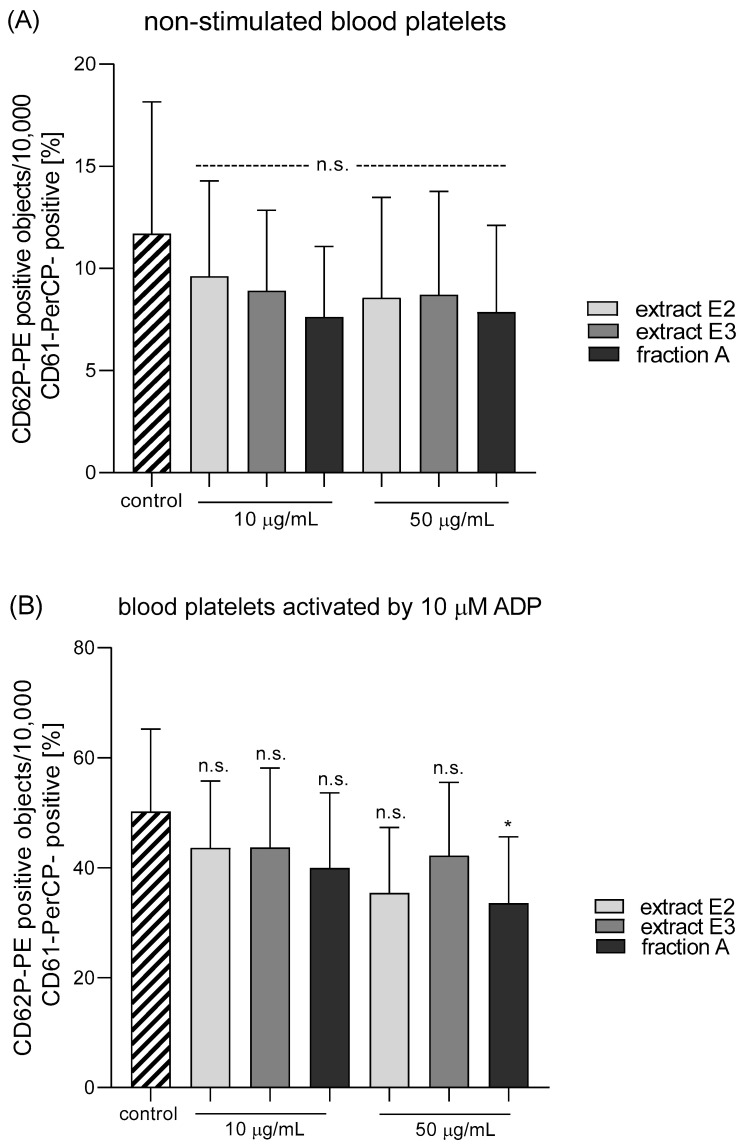

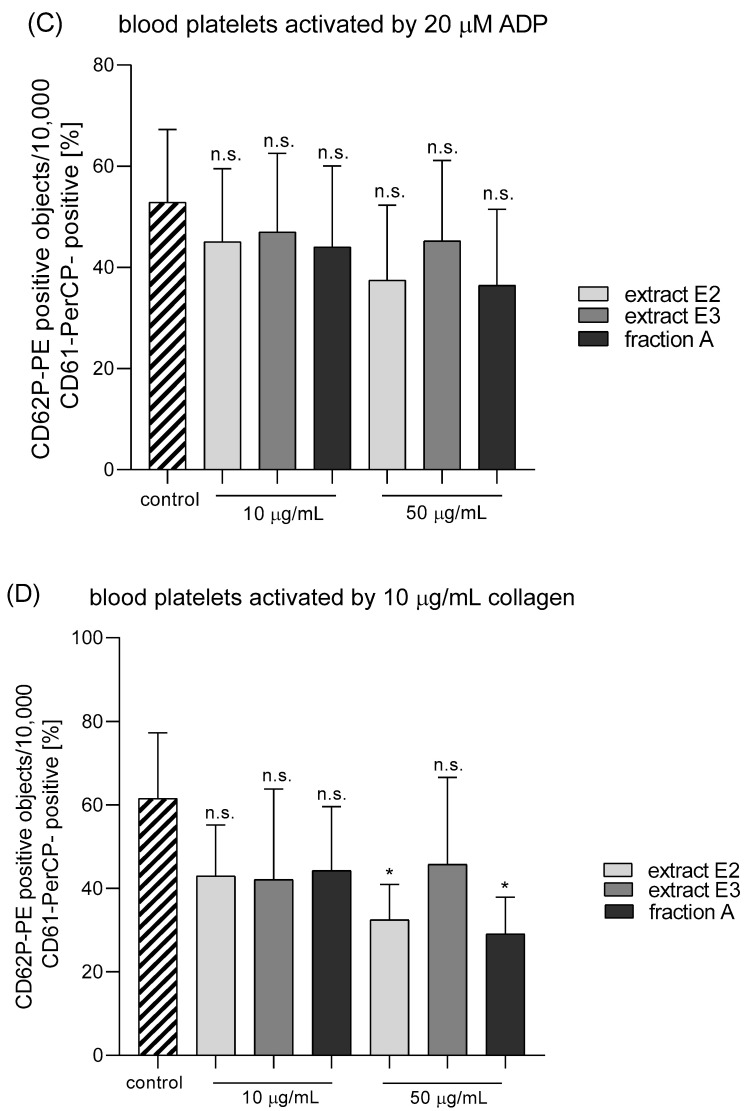
Effects of dandelion fruit preparations (E2, E3, and fraction A; concentrations 10 and 50 µg/mL) on the expression of P-selectin in resting (**A**) or agonist-stimulated blood platelets: 10 µM ADP (**B**), 20 µM ADP (**C**) and 10 µg/mL collagen (**D**) in whole blood samples. The blood platelets were distinguished based on the expression of CD61/PerCP. For each sample, 10,000 CD61-positive objects (blood platelets) were acquired. For the assessment of P-selectin expression, samples were labeled with fluorescently conjugated monoclonal antibody CD62P. Results are expressed as the percentage values of platelets expressing CD62P. Data are presented as the means ± SE of six healthy volunteers. n.s. *p* > 0.05; * *p* < 0.05 (vs. control platelets).

**Figure 11:**
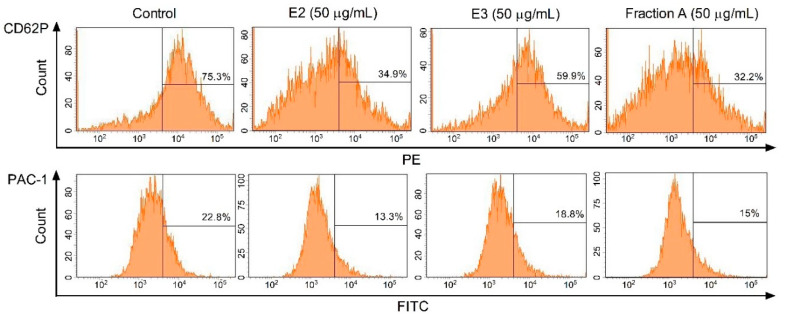
Effects of dandelion fruit preparations (E2, E3, and fraction A; concentration 50 µg/mL) on the expression of P-selectin and the active form of GPIIb/IIIa in platelets stimulated by 10 µg/mL collagen in whole blood samples. Figure demonstrates selected diagrams.

Three dandelion fruit preparations with the highest antioxidant and antiplatelet potential (extracts E2 and E3, and fraction A) were selected for subsequent flow cytometry analyses. The samples treated with extract E2 or fraction A demonstrated different blood platelet activation states compared with the untreated control samples (Figure 9 and Figure 10). For both above preparations, the effect was only evident at the higher test concentration (50 µg/mL), for example, E2 significantly reduced PAC-1 binding in platelets activated by 20 µM ADP or 10 µg/mL collagen (Figure 9C,D); it also significantly reduced CD62P expression on platelets activated by 10 µg/mL collagen (Figure 10D). In turn, extract E3 did not influence platelet P-selectin expression or GPIIb/IIIa expression for any of the four used models (Figure 9, Figure 10 and Figure 11).

**Figure 12:**
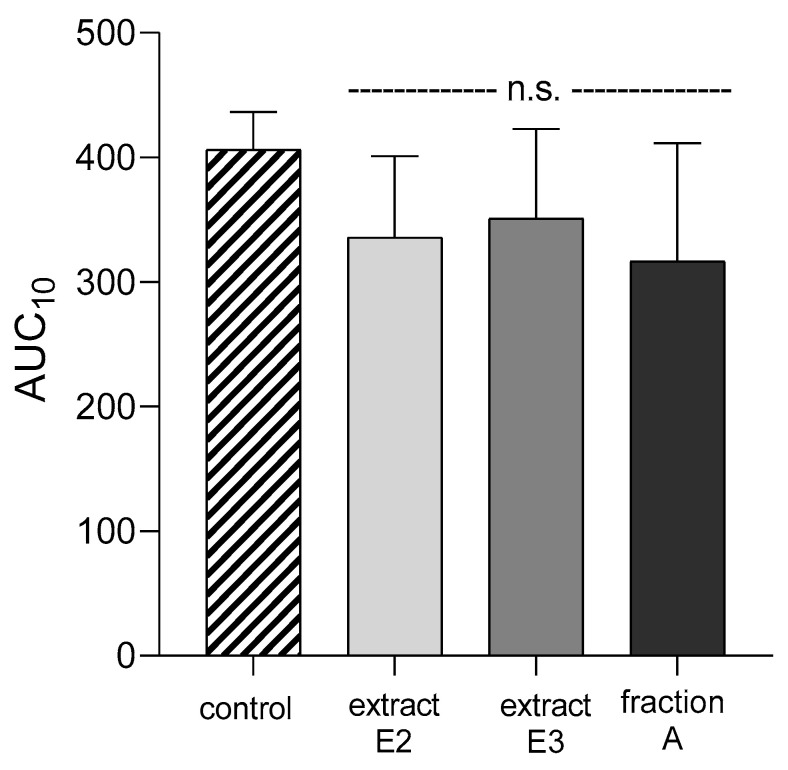
Effects of dandelion fruit preparations (E2, E3, and fraction A; concentration 50 µg/mL) on the T-TAS using the PL-chip in whole blood samples. Whole blood samples were analyzed by the T-TAS at the shear rates of 1000 s^−1^ on the PL-chips. The area under the curve (AUC_10_) in PL are shown as closed circles. Data represent the means ± SE of six healthy volunteers; n.s. *p* > 0.05, compared with control.

None of the dandelion fruit preparations changed the APTT, PT, TT, or AUC_10_ values measured by T-TAS for either human plasma or whole blood (Figure 12, Table 4).

### 2.5. Cytotoxicity against Blood Platelets

**Figure 13:**
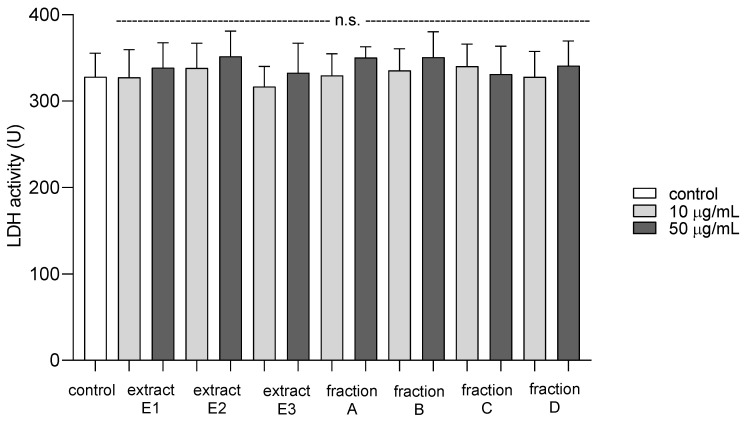
Cytotoxic effect of extracts (E1–E3) and flavonoid fractions (A–D) of dandelion fruits (concentrations: 10 and 50 µg/mL) on human blood platelets. Data are presented as means ± SD (*n* = 4); n.s. *p* > 0.05, compared with control.

To examine the toxicity of all dandelion fruit preparations on platelets, the extracellular LDH activity was measured. Compared to a control group, there was no significant difference in the viability of platelets after exposure to dandelion extracts (E1–E3) and fractions (A–D) at 10 and 50 µg/mL (Figure 13).

Table 5 compares the effects of three extracts (E1–E3) and four fractions (A–D) from dandelion fruits on the biological properties of plasma and blood platelets. It can be seen that extract E2 and fraction A demonstrated stronger antioxidant and antiplatelet properties than the other tested preparations (Table 5).

## 3. Discussion

The fruits of *Taraxacum officinale* are not as widely used in phytopharmacology as its other organs, and little is hence understood of their chemical composition and biological properties. In the continuation of our study on the dandelion, the current paper was aimed at the phytochemical analysis of *T. officinale* fruit extract and estimating its antiradical, antiplatelet, and antioxidant properties related to hemostasis. Efforts were also made to estimate the contribution of individual polyphenols on the reported biological activity of dandelion fruits. The idea behind the separation/fractionation process of methanolic extract of fruits (E1) was, on the one hand, to facilitate and broaden the metabolite identification (preliminary LC-MS analysis showed the presence of some unreported compounds in dandelion), and on the other hand, to test all extracts and fractions for antioxidant and antiplatelet activity, and conclude about the input of individual compounds/groups of compounds into biological potential and exerted effect of dandelion fruits. Previous research on the *T. officinale* plant identified high numbers of hydroxycinnamic acid derivatives [16,17,18]; therefore, the present study focused more on fractions enriched in flavonoid compounds, which are also an important component of the aerial parts, i.e., the leaves, flowers, and fruits of the dandelion.

With the use of HR-QTOF-MS analysis, about 30 secondary metabolites were identified in *T. officinale* fruit extract. The phytochemical profile was dominated by flavonoids and hydroxycinnamic acid derivatives (Table 1). Most of the detected polyphenols, such as caffeic acid esters and flavones, have already been described in dandelion species [13,19,20]; however, several flavone derivatives previously undescribed in the genus *Taraxacum* and the *Asteraceae* family were also identified. These included a series of flavone-flavone dimers (biflavones), particularly philonotisflavone (2’,8-biluteolin), as well as luteolin 3’-*O*-glucoside (Figure S1).

The C-C biflavones are a diverse group of phenolic compounds characterized by a variety of flavone units and linkage sites and have been found in many fruits, vegetables, and plants [21]. However, philonotisflavone has previously been known only from the gametophyte of a few bryophyte species, such as *Philonotis Fontana* [22] and *Bartramia pomiformis* [23]. Moreover, biflavonoids have so far been found in only one member of the *Asteraceae* family: *Saussurea eopygmaea* Hand.-Mazz. (*Carduoideae* subfamily) [24]. Several studies on biflavonoids, such as amentoflavone and ginkgetin, as well as on plant extracts enriched with these compounds, such as *Gingko biloba* leaf extract, have found them to demonstrate a wide range of cytotoxic, antimicrobial, antiviral, antioxidative, and anti-inflammatory activities [25].

To date, several luteolin glycosides have been identified in the *Taraxacum* genus, including two different isomers of luteolin glucoside (7-*O*- and 4’-*O*-glc) [13]; however, the present findings also indicate the presence of a new compound: luteolin 3’-*O*-glucoside. In addition, several flavonolignans (tricin-lignan conjugates) were observed, such as calquiquelignan D/E and salcolin A/B, which were recently described by Choi et al [26]. in an extract from the whole *T. officinale* plant.

Studies on the biological activity of dandelion fruit preparations began with the radical scavenging DPPH test. Five preparations (E1–E3, and fractions A,B) demonstrated significant antiradical activities (IC_50_ 0.06–0.42 mg/mL), while fractions C-D displayed relatively weak activity (IC_50_ > 1.3 mg/mL). No simple correlation between polyphenol content (TPC) and antiradical activity (TE and IC_50_ values) was observed, indicating a significant influence of qualitative factor. The DPPH test demonstrated the higher free radical scavenging effect of dandelion fruit flavonoids (E3 extract) compared with phenolic acids (E2 extract), even though E3 had significantly lower TPC than E2 (Table 2 and Table 3). Similarly, luteolin, the main component of the flavonoid extract (E3), exhibited 2-fold higher TE (2.0 TE, Fraction A, Table 3) than chicoric acid (1.1 TE) [16], the main component of phenolic acid extract. Moreover, the results show that the antiradical potential of the fruit extract is very little influenced by flavonoids lacking an active B-ring catechol moiety, as exemplified by the C-D fractions. The tricin-lignan conjugates and flavone aglycones (chrysoeriol, tricin, and apigenin), the main components of fractions C and D, lack or have modified (methylated or substituted) the catechol system in the B ring of the aglycone, which is known to enhance the antiradical property of phenolics [27].

The anti-oxidative, antiplatelet, and hemostasis related effects of dandelion fruit preparations were investigated using several experiments in vitro. Hemostasis is based on a series of strictly-regulated processes that enable platelet activation, vascular repair, and blood clotting. The ability of blood to clot prevents its excessive loss when the skin or blood vessels are broken. Although a key role in the clotting process is played by fibrinogen, which acts to transform soluble fibrinogen into insoluble fibrin, the process requires a cascade of consecutive biochemical reactions to produce a fibrin clot: each of the factors involved is activated by a previously-activated factor [28]. However, three discrete phases can be distinguished in the formation of a platelet plug: adhesion, activation, and aggregation of platelets. The adhesion phase starts when a blood vessel is damaged, and during this phase, platelets begin to adhere to the exposed elements of the subendothelial layer.

This stage is followed by platelet activation, which is stimulated by contact with an agonist such as ADP, collagen, or thrombin. During this stage, the platelet cytoskeleton undergoes reorganization and the platelets become less regular in shape, thus facilitating easier adhesion of successive layers. After activation by thrombin and then together with diacylglycerol lipase, phospholipase A_2,_ or phospholipase C, the platelets release arachidonic acid (AA) from their cell membrane phospholipids. AA is converted to cyclic prostaglandin peroxides (PGG_2_ and PGH_2_) by cyclooxygenase (COX), and then to thromboxane A_2_ (TXA_2_) by thromboxane A_2_ synthase. The prostaglandin peroxides form a range of substances including malondialdehyde (MDA), taking place via a non-enzymatic pathway, whereas pro-aggregation PGF_2a_ and PGE_2_ prostaglandins are formed by S-glutathione transfer. The PGG_2_ and PGH_2_ peroxides are converted to antiaggregatory PGD_2_ prostaglandins by PGD_2_ isomerase. The AA metabolic pathway leads to the production of mainly hydroxy acids and hepoxylins, a process catalyzed by 12-lipoxygenase. AA is converted into epoxyicatrienic acids by epoxygenase, interacting with cytochrome P450, and the AA in platelet blood may be converted to isoprostanes via a non-enzymatic route [28]. This phase is followed by aggregation, in which successive platelets adhere to a single layer of cells, resulting in the eventual formation of a platelet plug.

Physiological hemostasis is maintained by an equilibrium between the efficiency of the blood vessel wall, platelet, coagulation, and fibrinolysis system and the inhibitors regulating them. Any imbalance leads to excessive bleeding or clotting [29,30]. To prevent the formation of clots and blockages, antiplatelet drugs are typically introduced. Antiplatelet, otherwise known as anti-aggregation, drugs inhibit specific stages of platelet activation, i.e., adhesion or aggregation. Administration of rhodium, for example, may affect the synthesis of thromboxane A_2_, and influence the cyclic concentration of AMP (cAMP) or the total conversion of AA. Other antiplatelet drugs may block platelet membrane receptors such as acetylsalicylic acid or clopidogrel [31]; however, their use can cause various side effects.

Our previous publications demonstrated that in vitro tests using plasma and platelets, well reflecting the conditions in a living organism, are very helpful in finding substances with antioxidant and antiplatelet properties. In the current study, dandelion fruit preparations (E1–E3, A–D) showed various effects on oxidative stress in human plasma and blood platelets, as well as hemostatic properties of blood platelets.

The antiplatelet potential of the tested preparations, evaluated as an inhibitory effect on the adhesion of washed blood platelets to fibrinogen, was determined colorimetrically. Fractions A, B, and C demonstrated the greatest anti-adhesive activity in the two used models: (1) adhesion of thrombin-activated platelets to fibrinogen and (2) adhesion of ADP-activated platelets to fibrinogen. In addition, changes in hemostasis, such as the degree of platelet activation, occurring following exposure to the tested dandelion fruit preparation were identified in whole blood by T-TAS. T-TAS is a microchip-based flow chamber system that evaluates thrombogenicity in whole blood and may be used to assess the influence of anti-thrombotic preparations on blood platelet activation and coagulation reactions against a collagen or collagen/tissue thromboplastin-coated surface [32]. All selected dandelion fruit preparations (E2, E3, and fraction A) were found to demonstrate anti-coagulant potential against the platelets bound to collagen; however, this activity was not statistically different from untreated blood samples.

Platelet activation was also assessed using flow cytometry analysis of P-selectin expression (CD62P) and activation of GPIIb/IIIa complex (PAC-1 binding) in whole blood samples containing unstimulated platelets, and platelets stimulated by ADP or collagen. Flow cytometry can be used for both diagnoses and basic research, and in this case, it enabled the biological activity of blood platelets to be measured in the natural environment, i.e., immediately after blood collection. GPIIb/IIIa receptors are a good marker of blood platelet activation as the increasing number and change of their conformation during activation, aggregation, and adhesion [33]. Our findings confirm that the surface expression of the active form of GPIIb/IIIa on blood platelets decreases in the presence of extract E2 and fraction A (luteolin); also indicate significantly lower blood platelet adhesion in the presence of the dandelion fruit preparations, especially fraction A against washed blood platelets: we propose, therefore, that inhibition of platelet adhesion to fibrinogen may be associated with low expression of GPIIb/IIIa.

Another marker of blood platelet activation is P-selectin: a glycoprotein present in the alpha granules of unstimulated platelets. During activation, P-selectin is released with the alpha granules and can be seen on the surface of the platelet. Present findings indicate that P-selectin expression was reduced in whole blood following treatment with luteolin fraction (A) and hydroxycinnamic acids extract (E2). Finally, it appeared that luteolin demonstrated the best anti-platelet properties of the two preparations, which was observed not only in washed blood platelets but also in whole blood. Our observations are consistent with the literature: Benavente-Garcia and Castillo [34] reported that luteolin has antithrombotic activity, while Guerrero et al [35]. and Dell’Agli et al [36]. found it to have anti-platelet potential.

Platelet activation is associated with arachidonic acid metabolism. Our study demonstrates that all fruit flavonoid enriched preparations (E3, and A–D) reduced enzymatic lipid peroxidation in thrombin-activated platelets in vitro, as indicated by TBARS measurements. This finding suggests that dandelion fruit flavonoids can modulate blood platelet activation by interfering with the metabolism of arachidonic acid. No such change was observed for the other tested extracts, *viz.* E1 and E2, even at the highest used concentration, due to different chemical profiles—lack or low level of flavonoids.

In the anti-oxidative experiments, blood platelets and plasma were exposed to a hydroxyl radical donor (H_2_O_2_/Fe^2+^) and protective effects of dandelion preparations were measured. Four models were used to evaluate this activity: (1) plasma lipid peroxidation, (2) plasma protein carbonylation, (3) platelet lipid peroxidation, and (4) protein thiol oxidation or protein carbonylation in platelets treated with H_2_O_2_/Fe^2+^. It was found that extracts E1–E3 and fraction A (luteolin) demonstrated the best antioxidant properties. The results were therefore largely, but not entirely (an example of fraction B), consistent with the antiradical test with DPPH (E2-E3 extracts and luteolin fraction). In addition, a strong protective effect against the oxidation of plasma lipids and proteins exerted by hydroxycinnamic acids extract (E2), dominated by L-chicoric acid, is consistent with our previous publications [16,17].

The compounds extracted from the dandelion fruits appeared to be safe for use in the blood model, as none of the tested preparations (concentrations 10 and 50 µg/mL) demonstrated the cytotoxicity against platelets, measured as extracellular LDH activity. It is important to note that the concentrations of the tested extracts and fractions correspond with the physiological concentrations of phenolic compounds, including flavonoids, available after oral supplementation [37,38].

Flavonoids are metabolized by a two-phase process: phase I, based on hydroxylation and demethylation by cytochrome P 450 takes place in the liver, and phase II, based on O-methylation and coupling with glucuronic or sulfuric acid, in the intestine. These products are excreted with urine and bile; but must first pass through the intestinal-hepatic circulation, thus prolonging their elimination time and possible activity. The non-absorbent and bile-derived flavonoid metabolites are processed by the intestinal microflora, mainly in the large intestine. Bacterial enzymes can catalyze reactions such as the hydrolysis of glucuronides, sulfates, and glycosides, as well as dehydroxylation, demethylation, double bond reduction, and decomposition of the C-ring with the formation of phenolic acids, followed by their decarboxylation. Phenolic acids can also be absorbed, conjugated, or *O*-methylated in the liver and then excreted with the urine. Absorption may be relevant to total plasma antioxidant activity, as catecholytic acids show free radical scavenging activity [39]. Interestingly, various metabolites of phenolic compounds have been found as stronger inhibitors of platelet activation than their precursors [40].

In conclusion, the present study provides new information on *Taraxacum* metabolites, such as biflavonoid–philonotisflavone, and luteolin 3′-*O*-glucoside. From biological studies, it can be concluded that both the antioxidant and antiplatelet potential of dandelion fruit, demonstrated in several different in vitro experiments, was mainly due to luteolin (the main component of fraction A) and chicoric acid (the main component of E2 extract). We suggest that inhibition of platelet activation by fraction A may be associated with inhibition of receptor’s expression (including GPIIb/IIIa) and inhibition of arachidonic acid metabolism. By inhibiting platelet activation and oxidative stress, dandelion fruits and their polyphenols can be recognized as novel and valuable phytopharmacological agents. However, the mechanism of their antiplatelet and antioxidant properties remains unclear and required further studies. Additional studies, including in vivo experiments, are also needed to investigate the overall antioxidant and antiplatelet effects of dandelion fruits.

## 4. Materials and Methods

### 4.1. Chemical Reagents

Acetonitrile (isocratic grade and LC-MS grade), methanol (isocratic grade), and formic acid (98–100% purity) were purchased from Merck (Darmstadt, Germany). Dimethylsulfoxide (DMSO), thiobarbituric acid (TBA), hydrogen peroxide (H_2_O_2_), guanidine hydrochloride, bovine serum albumin (BSA), MS-grade formic acid, 1,1-diphenyl-2-picrylhydrazyl (DPPH), gallic acid, Folin-Ciocalteu reagent, and Trolox were acquired from Sigma-Aldrich (St. Louis, MO., USA). All other reagents were purchased from commercial suppliers, including POCH (Poland), Kselmed (Poland), Chrono-log (Poland), and Chempur (Poland). Ultrapure water was prepared in-house using a Milli-Q water purification system (Millipore, Milford, MA, USA). Fibrinogen was isolated from pooled human plasma, according to Doolittle [41]. The concentration was determined spectrophotometrically at 280 nm using an extinction coefficient of 1.55 for 1 mg/mL solution (final concentration of fibrinogen was 2 mg/mL).

### 4.2. Plant Material

Dandelion fruits were harvested in May 2017 on a farm located in south-eastern Poland (50°05’ N, 21°57’ E). The plant material was freeze-dried (Gamma 2–16 LSC, Christ, Osterode am Harz, Germany), pulverized (Grindomix GM200, Retsch, Haan, Germany), and stored in a refrigerator before extraction. A voucher specimen (TO-F-2017.05-1) is deposited at the Department of Biochemistry and Crop Quality of the Institute of Soil Science and Plant Cultivation–State Research Institute in Pulawy.

### 4.3. Preparation of Phenolic Extracts and Fractions from Dandelion Fruits

The finely-ground dandelion fruits (700 g) were defatted by extraction with *n*-hexane (4 L) under reflux (eight hours). The obtained defatted material (565 g) was twice extracted with 80% methanol (*v/v*; 4 L × 2; 12 h × 2) at 30 °C and sonicated (12 × 10 min) to enhance the extraction efficiency. The methanol extracts were filtered and combined, obtaining extract E1, which was then concentrated with a vacuum rotary evaporator (40 °C). The aqueous suspension of E1 (~50 g) was subjected to liquid-liquid extraction with ethyl acetate (0.75 L × 3). After evaporation of the organic solvent, flavonoid extract (E3) was freeze-dried to give 8.1 g of E3. The aqueous residue (~40 g) was found to contain large amounts of water-soluble primary metabolites (carbohydrates and amino acids) in addition to phenolics, as indicated by liquid chromatography-mass spectrometry (LC-MS) analysis. It was further purified by solid-phase extraction (SPE) on a short C18 column (12 × 5 cm, Cosmosil C18-PREP, 140μm, Nacalai Tesque Inc., Kyoto, Japan). The ballast components were washed with 4% methanol (*v/v*), and the compounds of interest were eluted with 80% methanol (*v/v*). After evaporation of the organic solvent, phenolic acid extract (E2) was lyophilized to give 9.5 g of E2.

The flavonoid extract (E3) was further fractionated isocratically on a Sephadex LH-20 (Sigma–Aldrich) column (80 × 2.8 cm i.d.) using 95% methanol (*v/v*) at a flow rate of 2.4 mL/min. The single sample capacity was 800 mg (dissolved in 10 mL of 95% methanol). The separation was monitored by LC-MS analyses. The five pooled fractions (I–V) were collected, concentrated, and freeze-dried to give 1290 mg of fraction I, 115 mg of fraction II, 850 mg of fraction III, and 480 mg of fraction IV. Due to their complex composition, or the presence of impurities, as indicated by LC-MS analysis (data not shown), the four fractions (I–IV) were further purified by high-performance liquid chromatography (HPLC) (Dionex, Sunnyvale, CA, USA) on a system equipped with a photodiode array detector (PDA-100) and FC 204 fraction collector (Gilson, Middleton, WI, USA). Separations were carried out on an Atlantis T3 C18 semi-preparative column (250 × 19 mm, 5 μm, Waters) at 40 °C with aqueous acetonitrile, containing 0.1% formic acid, at a flow rate of 5.5 mL/min as mobile phase. The conditions of the chromatographic run were individually optimized for each fraction (isocratic or gradient mode between 15–55% acetonitrile). The PDA detector was operated at 210 and 345 nm (5 nm bandwidth). The separation was monitored by LC-MS analyses. In total, four fractions (A–D) were collected, concentrated at 40 °C, and freeze-dried: the total yields were 1150 mg for fraction A, 95 mg for fraction B, 275 mg for fraction C, and 375 mg for fraction D. The extraction and fractionation process are illustrated in Figure S1.

### 4.4. Phytochemical Profiling

#### 4.4.1. Qualitative High-Resolution LC-MS Analysis

The dandelion fruit extracts (E1–E3) and fractions (A–D) were analyzed with a Thermo Ultimate 3000RS (Thermo Fischer Scientific, Waltham, MS, USA) chromatography system equipped with a diode array detector (DAD) and corona-charged aerosol detector (CAD), and coupled with a Bruker Impact II HD (Bruker, Billerica, MA, USA) quadrupole-time of flight (Q-TOF) mass spectrometer (MS). Chromatographic separations were carried out on an HSS C18 column (100 × 2.1 mm, 1.7 μm, Waters, Milford, MA, USA) at 40 °C. The injection volume was 3 μL. Mobile phase A was 0.1% (*v/v*) formic acid in MilliQ water, and mobile phase B consisted of acetonitrile containing 0.1% (*v/v*) of formic acid. The sample was separated using a linear gradient from 2 to 50% of solvent B in solvent A (0.4 mL/min, 13 min).

The UV absorbance was measured in the range of 200–600 nm (5 nm bandwidth). The acquisition frequency of both the DAD and CAD detector was set to 10 Hz. The MS analysis was performed in both ESI(–) and ESI(+) ion modes, using the following settings: scanning range 50–1800 *m/z*; negative ion capillary voltage 3.0 kV; positive ion capillary voltage 4.5 kV; dry gas flow 6 L/min; dry gas temperature 200 °C; nebulizer pressure 0.7 bar; collision RF 750 Vpp; transfer time 100 µs; prepulse storage time 10 µs. The collision energy was set to 20 eV. The results were calibrated internally with sodium formate injected into the ion source at the beginning of separation. Data processing was performed using Bruker DataAnalysis 4.3 software.

#### 4.4.2. Quantitative LC-UV Analysis of Flavonoids and Phenolic Acids

The flavonoid and phenolic acid contents in the dandelion extracts and fractions were determined using an ACQUITY UPLC system (Waters) equipped with a photodiode array detector (PDA) and a tandem quadrupole (TQD) mass spectrometer. Chromatographic separations were carried out on an HSS C18 column (100 × 2.1 mm, 1.7 μm, Waters), using parameters identical to those described in Section 4.4.1. The injection volume was 2.5 μL. The UV spectra were recorded within the range of 190–490 nm (3.6 nm resolution).

The lyophilized samples were dissolved in 80% methanol at a concentration of 2 mg DW/mL (two replicates were prepared for each sample) and appropriately diluted before chromatographic analyses. Flavonoids were detected at UV_345nm_ and phenolic acids at UV_320nm_. Quantitative determinations were based on an external standard method, using two group standards: luteolin and L-chicoric acid (the main flavonoid and phenolic acid of dandelion fruits, respectively). Both reference compounds had been previously isolated by our laboratory and their purity was determined by LC-MS analysis: L-chicoric acid (95%) [16], and luteolin (99%).

Both the luteolin and L-chicoric acid calibration curves were prepared in six concentrations, ranging from 0.5 to 150 μg/mL, and showed good linearity (R^2^ ≥ 0.999). Three injections were performed for each sample/standard working solution. Quantitative results were expressed as mg standard (L-chicoric acid or luteolin) equivalents (eq)/g of extract/fraction.

### 4.5. Determination of Total Phenolic Content (TPC)

The total phenolic content in all samples was determined with the Folin-Ciocalteu assay, as described previously [16]. Briefly, 100 µL of F-C reagent was added to 1600 µL of an appropriately diluted sample (6–25 µg/mL) or gallic acid standard solution (six concentrations in the range between 0.5–8 µg/mL). After adding 300 µL of Na_2_CO_3_ (20% *w/v*), the mixture was incubated in a water bath (40 °C) for 30 min. The absorbance was measured at 765 nm against a blank sample using an Evolution 260 Bio spectrophotometer (Thermofisher Scientific). The TPC of samples was read from the linear curve for gallic acid (R^2^ > 0.999) and expressed as milligrams of gallic acid equivalents (mg GAE/g DW).

### 4.6. DPPH Free Radical Scavenging Activity

The radical scavenging activity of the extracts and fractions against the DPPH free radical was determined according to Brand-Williams [42], with slight modifications as described by Lis et al [17]. Briefly, 1900 µL of DPPH methanol solution (100 µM) was mixed with 100 µL of the sample (four different concentrations in the range between 10–500 µg/mL) or Trolox solution (Sigma-Aldrich; five different concentrations in the range between 10–200 µg/mL) in a cuvette. After 30 min, the absorbance was measured against methanol (blank) at 517 nm using the Evolution 260 Bio spectrophotometer (Thermofisher Scientific). The percentage of absorbance inhibition was calculated from the equation:

Inhibition (%) = 100 × [(A_blank_ − A_sample_)/A_blank_], where A_blank_ and A_sample_ are the absorbance values of the blank and test samples at *t* = 30 min, respectively.

To calculate the Trolox Equivalent (TE) of samples on DPPH, the slope of the sample linear curve, i.e., the absorbance inhibition (%) vs. concentration (µg/mL), was divided by the slope of the standard linear curve.

The IC_50_ value of extracts and fractions, defined as the concentration of sample necessary to cause 50% inhibition was determined from the sample linear curves as absorbance inhibition (%) vs. concentration (µg/mL), with Trolox used as a positive control.

### 4.7. Blood Platelets and Plasma Isolation

Human blood was obtained from a Medical Center in Lodz (Poland), and the biological material came from regular, non-smoking/non-drinking alcohol and medication-free donors. The blood was collected into tubes with citrate/phosphate/dextrose/adenine (CPDA) anticoagulant. All experiments were approved by the University of Lodz Committee for Research on Human Subjects and carried out under permission number 2/KBBN-UŁ/II/2016.

Plasma and blood platelets were isolated from fresh human blood by differential centrifugation as described previously [16]. The platelet pellet was suspended in modified Tyrode’s buffer (pH 7.4). The number of platelets in suspensions used in the experiments was determined at 800 nm using a UV-Visible Helios α spectrophotometer (Unicam) according to Walkowiak et al [43].; the amount was found to be 1.5–2.5 × 10^8^/mL. The protein concentration was calculated by measuring the absorbance of tested samples at 280 nm according to Whitaker and Granum [44]. Each sample (both plasma and blood platelets) taken for testing was obtained from different subjects and could be regarded as an independent trial.

### 4.8. Incubation of Plasma, Blood Platelets, and Whole Blood with Plant Extracts and Fractions

Stock solutions of the *T. officinale* L. fruit extracts and fractions were prepared with 50% DMSO (*v/v*). The final concentration of DMSO in test samples was lower than 0.05%, and its effects were determined in all experiments. The extracts and fractions were then taken for use in different models to evaluate the antithrombotic properties. Briefly:

The plasma was pre-incubated for five minutes at 37 °C with dandelion extracts (E1–E3) and fractions (A–D) at two concentrations, 10 and 50 µg/mL, and then treated with 4.7 mM H_2_O_2_/3.8 mM Fe_2_SO_4_/2.5 mM EDTA (25 min, at 37 °C).

The blood platelets were incubated for 30 min at 37 °C with dandelion extracts (E1–E3) and fractions (A–D) at final concentrations of 10 and 50 µg/mL.

The blood platelets were pre-incubated for five minutes at 37 °C with dandelion extracts (E1–E3) and fractions (A–D) at two concentrations, 10 and 50 µg/mL, and then treated with 4.7 mM H_2_O_2_/3.8 mM Fe_2_SO_4_/2.5 mM EDTA (25 min at 37 °C).

The blood platelets were pre-incubated (25 min at 37 °C) with dandelion extracts (E1–E3) and fractions (A–D) at two concentrations, 10 and 50 µg/mL, and then treated with thrombin at a final concentration of 5 U/mL (5 min at 37 °C).

Whole blood was incubated (30 min at 37 °C) with dandelion extracts (E2 and E3) and fraction A at final concentrations of 10 and 50 µg/mL.

### 4.9. Parameters of Blood Platelet Activation

#### 4.9.1. Platelet Adhesion to Fibrinogen

Adhesion of blood platelets to fibrinogen was determined colorimetrically by measuring acid phosphatase activity [45]. Blood platelets were incubated in microtiter plates that had been precoated with fibrinogen, then nonadherent platelets were washed out and dissolved with Triton X-100. The details of the procedure were described previously [16,17]. Finally, 2M NaOH was added and the *p*-nitrophenol produced was measured at 405 nm, using SPECTROstar Nano Microplate Reader (BMG LABTECH, Germany). A control group was platelets without the addition of plant preparations; and its absorbance was expressed as 100%.

#### 4.9.2. Flow Cytometry

Changes in the activation and reactivity of resting and stimulated blood platelets were studied in the whole blood model [46]. Fresh blood samples (150 µL) were incubated with dandelion fruit preparations (30 min., 25 °C), and then stimulated with 10 and 20 µM ADP for 15 min at room temperature (RT), or collagen (10 µg/mL, 15 min, RT). After incubation, the samples were diluted tenfold (1:9) in sterile PBS with Mg^2+^ and stained with anti-CD61/PerCP, anti-CD62/PE, or PAC-1/FITC antibodies (30 min, RT, in the dark). Appropriate isotype controls were then prepared: the resting blood samples were stained with anti-CD61/PE and isotype control antibodies marked with FITC or PE. Finally, all samples were fixed with 1% CellFix (60 min, 37 °C).

The platelets were counted using an LSR II Flow Cytometer (Becton Dickinson, San Diego, CA, USA) based on the fluorescence of 10,000 platelets (CD61/PerCP positive objects). The platelets were distinguished from other blood cells by a forward light scatter (FCS) vs. side light scatter (SSC) plot on a log/log scale (first gate) and by positive staining with monoclonal anti-CD61/PerCP antibodies (second gate). The percentages of CD62P-positive and PAC-1-positive platelets were calculated relative to the total number of platelets (CD61-positive cells) presented in each sample. Non-specific antibody binding was determined using the isotype control antibodies IgG1/PE and IgM/FITC. All results were analyzed using BD FACSDiva software (Becton Dickinson, San Diego, CA, USA).

### 4.10. Parameters of the Coagulation Process

#### 4.10.1. Thrombin Time (TT) Measurement

Plasma (50 µL) was incubated in a measuring cuvette (1 min., 37 °C), then a 100 µL of thrombin at the final concentration of 5 U/mL was added. The TT was measured using an Optic Coagulation Analyser (Kselmed, Poland). The procedure was carried out as described by Malinowska et al [47]. Time until clot formation was measured, the results are given in seconds. Samples were tested in duplicate.

#### 4.10.2. Prothrombin Time (PT) Measurement

Plasma (50 µL) was incubated in a measuring cuvette (2 min., 37 °C), then a 100 µL of Dia-PT liquid (commercial preparation) was added. The PT was measured using an Optic Coagulation Analyser (Kselmed, Poland). The procedure was carried out as described by Malinowska et al [47]. Time until clot formation was measured, the results are given in seconds. Samples were tested in duplicate.

#### 4.10.3. Activated Partial Thromboplastin Time (APTT) Measurement

Fifty microliters of Dia-PTT liquid (commercial preparation) were added to 50 µL of human plasma, followed by incubation at 37 °C (3 min). Afterward, a 50 µL of 25 mM CaCl_2_ was added. The APPT was measured using an Optic Coagulation Analyser (Kselmed, Poland). The procedure was carried out as described by Malinowska et al [47]. Time until clot formation was measured, the results are given in seconds. Samples were tested in duplicate.

#### 4.10.4. Total Thrombus Formation Analysis System (T-TAS^®^)

T-TAS^®^ was used to analyze the thrombus formation process under flow conditions. Platelet thrombus formation was measured using the PL-chip microchip coated with collagen. Whole blood (400 µL) anticoagulated with BAPA (benzylsulfonyl-D-arginyl-prolyl-4-amidinobenzylamide) was incubated with the tested fractions (30 min., 37 °C). Subsequently, samples (340 µL) were transferred to the PL-chip. The results were taken as AUC_10_ i.e., Area Under the Curve [48].

### 4.11. Cytotoxicity

The cytotoxicity of dandelion fruit preparations against blood platelets was examined by measuring extracellular LDH activity. Firstly, the 270 µL of 0.1 M phosphate buffer, 10 µL of supernatant, and 10 µL of nicotinamide-adenine-dinucleotide (NADH) were added to the microtiter plate and then incubated (20 min., 25 °C). Thereafter, 10 µL of pyruvate was added and the absorbance was immediately measured at 340 nm using a SPECTROstar Nano Microplate Reader (BMG LABTECH, Germany). The measurement was carried out for 10 min, and it was repeated every minute [49].

### 4.12. Parameters of Oxidative Stress

#### 4.12.1. Lipid Peroxidation Measurement

Lipid peroxidation was determined by measuring the concentration of thiobarbituric acid reactive substances (TBARS). The procedure was carried out according to Bartosz [4]. After incubation of plasma with dandelion preparations, an equal volume of cold 15% (*v/v*) trichloroacetic acid in 0.25 M HCl and 0.37% (*v/v*) thiobarbituric acid in 0.25 M HCl were transferred to the mixture. Then, the samples were incubated in the boiling water bath for 10 min, followed by cooling in an ice bath and centrifugation (10,000× *g*, 15 min, 18 °C) [4,50]. Absorbance of the colored product was measured at 535 nm using a SPECTROstar Nano Microplate Reader (BMG LABTECH, Germany). The TBARS concentration was calculated using the molar extinction coefficient (ε = 156,000 M^−1^ cm^−1^). The results were expressed as nmol/mL of plasma, or nmol/10^8^ blood platelets.

#### 4.12.2. Carbonyl Group Measurement

The level of protein carbonyl groups in plasma was measured by the addition of 2,4-dinitrophenylhydrazine (DNPH), which in the dark binds with this functional group. The resulting colored compound was assayed spectrophotometrically at 375 nm using a SPECTROstar Nano Microplate Reader (BMG LABTECH, Germany). The procedure was carried out according to Levine et al [51]. and Bartosz [4]. Carbonyl group concentration was calculated based on the molar extinction coefficient (ε = 22,000 M^−1^ cm^−1^). The results were expressed as nmol/mg of plasma protein, or nmol/mg of platelet protein.

#### 4.12.3. Thiol Group Determination

Thiol group level was measured using Ellman’s reagent, i.e., 5,5′-dithiobis-(2-nitrobenzoic) acid (DTNB). The procedure was carried out according to Ando and Steiner [52,53]. The resulting colored compound was assayed spectrophotometrically at 412 nm using a SPECTROstar Nano Microplate Reader (BMG LABTECH, Germany). Finally, the thiol group concentration was calculated based on the molar extinction coefficient (ε = 136,000 M^−1^ cm^−1^). The results were expressed as nmol/mg of plasma protein, or nmol/mg of platelet protein.

### 4.13. Data analysis

Data distribution was checked by normal probability plots, and the homogeneity of variance by Levene’s test. Differences within and between groups were assessed by the Kruskal-Wallis test; for the sake of clarity, only the differences between the tested preparations and the control/control positive were marked. Additionally, in Table 2 and Table 3, the data was marked with color code, for easy visualization of the differences between the preparations, generated with the use of the Quick Analysis tool (Microsoft Excel). Results are presented as means ± SD. Significance was considered at *p* < 0.05. To eliminate uncertain data, the Q-Dixon test was performed.

## Figures and Tables

**Table 1:** UHPLC-QTOF-MS/MS data of metabolites identified in the methanol extract (E1) of *Taraxacum officinale* L. fruits.

No	Identity	RT (min)	Formula	UV_max_ (nm)	Error (ppm)	mσ	Observed [M − H]^−^	Major Fragments (%)
1	unidentified	1.10	C_13_H_9_N_4_O_6_	260	−5.2	28.6	317.0544	225.0072 (23), 164.9851 (3)
2	di-hexose (sucrose)	1.27	C_13_H_23_O_13_	220	1.6	2.3	387.1138 [M + HCO_2_H − H]^−^, 341.1085	179.0563 (17), 161.0449 (5)
3	tri-hexose (raffinose)	1.27	C_19_H_33_O_18_	220	1.1	7.1	549.1666 [M + HCO_2_H − H]^−^, 503.1610	179.0563 (8), 341.1085 (5)
4	unidentified	1.63	C_9_H_11_N_2_O_6_	275	0.6	5.7	243.0621	-
5	unidentified	3.39	C_10_H_11_N_4_O_6_	262	1.2	9.9	283.0681	151.0256 (100)
6	caffeoyl-di-*O*-hexoside	4.08	C_22_H_29_O_16_	290br	1.8	6.7	549.1455 [M + HCO_2_H − H]^−^	341.0875 (100), 179.0349 (10)
7	di-hydroxy-benzoic acid hexoside	4.52	C_13_H_15_O_9_	305	0.5	15.2	315.0720	153.0186 (100)
8	caftaric acid	4.78	C_13_H_11_O_9_	328, 295sh	−0.3	15.7	311.0410	179.0348 (100), 135.0438 (51), 149.0079 (15)
9	unidentified	4.97	C_15_H_20_NO_6_	328, 295sh	2.6	16.1	310.1288 [M + HCO_2_H − H]^−^	250.1084 (100), 161.0237 (4), 179.0356 (2)
10	caffeoyl-*O*-hexoside	5.48	C_15_H_17_O_9_	320, 290sh	−0.3	15.8	341.0879	179.0351 (100), 135.0444 (16)
11	5-*O*-caffeoylquinic acid *	5.59	C_16_H_17_O_9_	325, 295sh	0.3	5.1	353.0877	191.0562 (100)
12	caffeic acid	6.11	C_9_H_7_O_4_	323, 295sh	−0.8	7.6	179.0351	135.0440 (100)
13	unidentified	6.37	C_18_H_27_O_9_	308br	−0.3	28.9	387.1664	207.1029 (15)
14	luteolin-*O*-di-hexoside	6.95	C_27_H_29_O_16_	255, 345	2.7	6.8	609.1445	285.0394 (12)
15	L-chicoric acid	7.38	C_22_H_17_O_12_	329, 295sh	1.8	5.8	473.0727	179.0354 (100), 293.0306 (49), 311.0412 (44), 149.0088 (33), 219.0301 (26)
16	luteolin-7-*O*-glucoside	7.76	C_21_H_19_O_11_	255, 267, 345	−0.9	19.7	447.0937	285.0408 (96)
17	3,5-di-caffeoylquinic acid	8.26	C_25_H_23_O_12_	328, 295sh	1.2	4.5	515.1189	353.0871 (100), 191.0557 (26), 179.0347 (12)
18	luteolin-4’-*O*-glucoside	8.50	C_21_H_19_O_11_	269, 337	−1.6	18.1	447.0937	285.0408 (100)
19	luteolin-3’-*O*-glucoside #	8.85	C_21_H_19_O_11_	267, 337	−2.0	27.4	447.0942	285.0407 (100)
20	taraxinic acid-1’-*O*-glucoside	9.01	C_22_H_29_O_11_	220	−0.5	4.0	469.1718 [M + HCO_2_H − H]^−^	261.1135 (100), 217.1235 (59)
21	luteolin	10.23	C_15_H_9_O_6_	255, 267, 345	0.3	3.1	285.0404	199.0399 (4), 217.0506 (3), 241.0505 (3)
22	philonotisflavone #	10.23	C_30_H_17_O_12_	255, 342	−1.5	5.7	569.0734	391.0469 (8), 433.0574 (4), 459.0371 (3)
23	methyltricetin	10.27	C_16_H_11_O_7_	267, 347	0.4	15.0	315.0509	300.0274 (100), 272.0322 (7)
24	bi-flavone (luteolin-luteolin) #	10.52	C_30_H_17_O_12_	255, 345	−0.2	20.7	569.0727	417.0610 (10), 285.0411 (2)
25	bi-flavone (luteolin-apigenin) #	10.93	C_30_H_17_O_11_	257, 345	−2.3	25.2	553.0789	391.0478 (13), 433.0572 (3), 459.0370 (3)
26	bi-flavone (luteolin-apigenin) #	11.09	C_30_H_17_O_11_	257, 345	−1.4	6.4	553.0791	401.0682 (19)
27	bi-flavone (luteolin-chrysoeriol) #	11.28	C_31_H_19_O_12_	257, 343	−0.9	9.3	583.0891	431.0781 (11)
28	apigenin	11.32	C_15_H_9_O_5_	267, 337	−1.6	9.7	269.0460	225.0562 (10)
29	bi-flavone (luteolin-chrysoeriol) #	11.38	C_31_H_19_O_12_	257, 343	−1.0	7.6	583.0892	431.0779 (11)
30	flavonolignan (tricin-lignan (*m/z* 170) conjugate) #	11.50	C_26_H_23_O_10_	269, 338	2.7	1.7	495.1283, 541.1340 [M + HCO_2_H − H]^−^	329.0658 (100), 447.1076 (8)
31	tricin	11.54	C_17_H_13_O_7_	255, 267, 351	−1.7	0.5	329.0672	299.0204 (100), 314.0438 (71), 271.0256 (10)
32	chrysoeriol	11.62	C_16_H_11_O_6_	251, 267, 345	−2.2	7.0	299.0568	284.0333 (100), 256.0384 (13)
33	flavonolignan (salcolin A/B)	11.62	C_27_H_25_O_11_	271, 337	2.7	3.4	525.1388, 571.1445 [M + HCO_2_H − H]^−^	329.0659 (100), 314.0426 (14), 195.0659 (10), 165.0551 (7)
34	flavonolignan (tricin-lignan (*m/z* 170) conjugate) #	11.77	C_26_H_23_O_10_	271, 337	2.4	4.3	495.1285, 541.1342 [M + HCO_2_H − H]^−^	329.0659 (100), 314.0425 (24), 135.0441 (4)
35	flavonolignan (salcolin A/B)	11.94	C_27_H_25_O_11_	271, 337	2.1	1.4	525.1391, 571.1448 [M + HCO_2_H − H]^−^	329.0660 (100), 314.0425 (14), 195.0659 (9), 165.0551 (6)
36	flavonolignan (tricin derivative) #	12.07	C_37_H_35_O_14_	271, 340	2.7	18.1	703.2013, 749.2069 [M + HCO_2_H − H]^−^	329.0657 (100), 373.1282 (41), 673.1911 (30), 343.1178 (18), 685.1909 (16)
37	apometzgerin	12.12	C_17_H_13_O_7_	269, 335	−2.5	2.2	329.0675	314.0441 (100), 299.0206 (95), 271.0257 (11)
38	flavonolignan (tricin-lignan (*m/z* 194) conjugate) #	12.33	C_27_H_23_O_11_	271, 327	1.9	11.2	523.1236, 569.1291 [M + HCO_2_H − H]^−^	329.0659 (100), 314.0422 (6)
39	flavonolignan (tricin derivative) #	12.43	C_37_H_35_O_14_	271, 340	2.8	14.1	703.2013, 749.2069 [M + HCO_2_H − H]^−^	329.0658 (100), 373.1286 (53), 673.1913 (20), 343.1178 (17), 685.1911 (5)

* the identity of the underlined metabolites was confirmed with authentic isolated compounds. # metabolite reported for the first time in dandelion (*Taraxacum officinale* L.).

**Table 2:** Comparison of phytochemical profile and phenolic content in tested extracts (E1–E3) and fractions (A–D) of dandelion fruits.

No	Compound	Content [mg Standard eq/g DW] (Mean ± SD)						
		E1 (Total Extract)	E2 (Phenolic Acid Extract)	E3 (Flavonoid Extract)	Fr A (Luteolin)	Fr B (Philonotisflavone)	Fr C (Flavonolignans)	Fr D (Flavone Aglycones)
1	unidentified	+ ^a^	+	ND ^b^	ND	ND	ND	ND
2	di-hexose (sucrose)	+	+	+	ND	ND	ND	ND
3	tri-hexose (raffinose)	+	+	+	ND	ND	ND	ND
4	unidentified	+	+	+	ND	ND	ND	ND
5	unidentified	+	+	ND	ND	ND	ND	ND
6	caffeoyl-di-*O*-hexoside	6.02 ± 0.14	27.63 ± 0.20	ND	ND	ND	ND	ND
7	di-hydroxy-benzoic acid hexoside	+	+	ND	ND	ND	ND	ND
8	caftaric acid	4.97 ± 0.33	ND	ND	ND	ND	ND	ND
9	unidentified	+	+	+	ND	ND	ND	ND
10	caffeoyl-*O*-hexoside	1.02 ± 0.03	4.68 ± 0.26	ND	ND	ND	ND	ND
11	5-*O*-caffeoylquinic acid	21.97 ± 0.56	92.50 ± 1.23	1.88 ± 0.06	ND	ND	ND	ND
12	caffeic acid	1.41 ± 0.03	2.60 ± 0.08	9.19 ± 0.14	ND	ND	ND	ND
13	unidentified	+	ND	+	ND	ND	ND	ND
14	luteolin-*O*-di-hexoside	++ ^c^	2.42 ± 0.05	ND	ND	ND	ND	ND
15	L-chicoric acid	104.60 ± 3.33	384.40 ± 4.43	1.41 ± 0.10	ND	ND	ND	ND
16	luteolin-7-*O*-glucoside	1.42 ± 0.06	1.81 ± 0.05	6.13 ± 0.09	ND	ND	ND	ND
17	3,5-di-caffeoylquinic acid	7.54 ± 0.26	18.75 ± 0.50	17.19 ± 0.21	ND	ND	ND	ND
18	luteolin-4’-*O*-glucoside	1.00 ± 0.03	0.76 ± 0.04	3.85 ± 0.08	ND	ND	ND	ND
19	luteolin-3’-*O*-glucoside	0.64 ± 0.03	ND	4.46 ± 0.09	ND	ND	ND	ND
20	taraxinic acid-1’-*O*-glucoside	+	ND	+	ND	ND	ND	ND
21	luteolin	36.03 ± 1.12	ND	203.89 ± 2.58	985.86 ± 13.50	ND	ND	ND
22	philonotisflavone		ND		++	472.21 ± 1.51	ND	ND
23	methyltricetin		ND		8.56 ± 0.20	ND	ND	ND
24	bi-flavone (luteolin-luteolin)	0.85 ± 0.02	ND	3.73 ± 0.02	ND	ND	ND	ND
25	bi-flavone (luteolin-apigenin)	++	ND	++	ND	3.80 ± 0.22	ND	ND
26	bi-flavone (luteolin-apigenin)	++	ND	0.35 ± 0.03	ND	2.60 ± 0.09	ND	ND
27	bi-flavone (luteolin-chrysoeriol)	++	ND	1.50 ± 0.05	ND	++	ND	ND
28	apigenin	0.37 ± 0.04	ND	2.88 ± 0.18	ND	ND	ND	69.60 ± 1.10
29	bi-flavone (luteolin-chrysoeriol)	++	ND	1.20 ± 0.08	ND	5.44 ± 0.08	ND	ND
30	flavonolignan (tricin-lignan (*m/z* 170) conjugate)	++	ND	++	ND	ND	87.03 ± 5.18	ND
31	tricin	6.53 ± 0.17	ND	37.13 ± 0.41	ND	ND	ND	799.09 ± 8.19
32	chrysoeriol		ND		ND	ND	ND	
33	flavonolignan (salcolin A/B)	0.75 ± 0.01	ND	4.97 ± 0.12	ND	ND	107.28 ± 6.60	ND
34	flavonolignan (tricin-lignan (*m/z* 170) conjugate)	0.77 ± 0.01	ND	4.57 ± 0.14	ND	ND	96.93 ± 5.80	ND
35	flavonolignan (salcolin A/B)	0.89 ± 0.03	ND	6.02 ± 0.21	ND	ND	109.89 ± 6.39	ND
36	flavonolignan (tricin derivative)	++	ND	++	ND	ND	4.37 ± 0.42	ND
37	apometzgerin	++	ND	++	ND	ND	ND	18.01 ± 0.80
38	flavonolignan (tricin-lignan (*m/z* 194) conjugate)	++	ND	0.62 ± 0.01	ND	ND	11.74 ± 0.67	ND
39	flavonolignan (tricin derivative)	++	ND	++	ND	ND	++	ND
	Total caffeic acid derivatives	147.53 ± 4.68	530.56 ± 6.70	29.67 ± 0.51	ND	ND	ND	ND
	Total flavonoids	48.48 ± 1.52	4.99 ± 0.14	281.30 ± 4.03	994.42 ± 13.70	484.05 ± 1.90	417.24 ± 25.06	886.71 ± 10.09
	Total phenolic compounds	196.01 ± 6.20	535.55 ± 6.84	310.97 ± 4.60	994.42 ± 13.70	484.05 ± 1.90	417.24 ± 25.06	886.71 ± 10.09
	Total phenolic content (mg GAE/g DW)	187.70 ± 0.22	447.58 ± 2.21	377.42 ± 1.77	879.55 ± 2.76	516.13 ± 3.31	384.27 ± 2.21	631.71 ± 2.95

^a^ +, present but not quantified. ^b^ ND, not detected. ^c^ ++, concentration below the lower limit of quantification (LLOQ). Color code generated with the Quick Analysis tool (Microsoft Excel) indicates the level of metabolite content in tested preparations (green (higher level)→white (medium)→red (lower)).

**Table 3:** Antioxidant activity of extracts (E1–E3) and flavonoid fractions (A–D) of dandelion fruits against DPPH free radical (mean ± SD, *n* = 3) ^a,b^.

Sample	Trolox Equivalents (TE)	IC_50_ (mg DW/mL)
E1 (total extract)	0.26 ± 0.00b	0.424 ± 0.01d
E2 (phenolic acid extract)	0.48 ± 0.00c	0.215 ± 0.01c
E3 (flavonoid extract)	0.55 ± 0.03d	0.202 ± 0.01c
Fr A (luteolin)	2.01 ± 0.01g	0.055 ± 0.00a
Fr B (philonotisflavone)	1.09 ± 0.01f	0.099 ± 0.00b
Fr C (flavonolignans)	0.06 ± 0.00a	1.368 ± 0.04e
Fr D (flavone aglycones)	0.05 ± 0.00a	1.789 ± 0.04f
Trolox	1.00e	0.113 ± 0.00b

^a^ Within each column, different letters (a–g) indicate significant differences in means (*p* < 0.05). ^b^ Color code generated with the Quick Analysis tool (Microsoft Excel) indicates the level of activity of the tested preparations (red (high activity)→white (medium)→ green(low)).

**Table 4:** Effects of extracts (E1–E3) and flavonoid fractions (A–D) of dandelion fruits (concentrations 10 and 50 µg/mL) on the coagulation times of human plasma (APTT, PT, and TT). Data are presented as means ± SD (*n* = 10).

Sample Name	Tested Concentration (µg/mL)	(TT)	(PT)	(APTT)
			Mean ± SD	
control	0	15.5 ± 1.2 ^n.s.^	15.0 ± 0.6 ^n.s.^	43.1 ± 5.2 ^n.s.^
E1 (total extract)	10	15.5 ± 1.6 ^n.s.^	14.9 ± 0.5 ^n.s.^	43.5 ± 4.8 ^n.s.^
	50	15.6 ± 1.6 ^n.s.^	14.8 ± 0.5 ^n.s.^	43.4 ± 5.2 ^n.s.^
E2 (phenolic acid extract)	10	15.3 ± 1.5 ^n.s.^	14.8 ± 0.6 ^n.s.^	42.6 ± 4.4 ^n.s.^
	50	15.4 ± 1.5 ^n.s.^	14.9 ± 0.6 ^n.s.^	42.9 ± 4.7 ^n.s.^
E3 (flavonoid extract)	10	15.5 ± 1.4 ^n.s.^	14.7 ± 0.6 ^n.s.^	43.1 ± 4.5 ^n.s.^
	50	15.4 ± 1.5 ^n.s.^	15.0 ± 0.4 ^n.s.^	43.3 ± 4.5 ^n.s.^
Fr A (luteolin)	10	15.5 ± 1.3 ^n.s.^	14.9 ± 0.4 ^n.s.^	42.8 ± 4.3 ^n.s.^
	50	15.3 ± 1.5 ^n.s.^	14.9 ± 0.5 ^n.s.^	42.5 ± 4.4 ^n.s.^
Fr B (philonotisflavone)	10	15.5 ± 1.4 ^n.s.^	15.1 ± 0.7 ^n.s.^	42.5 ± 4.3 ^n.s.^
	50	15.3 ± 1.3 ^n.s.^	14.9 ± 0.7 ^n.s.^	43.1 ± 4.4 ^n.s.^
Fr C (flavonolignans)	10	15.4 ± 1.3 ^n.s.^	15.0 ± 0.6 ^n.s.^	42.3 ± 4.5 ^n.s.^
	50	15.7 ± 1.3 ^n.s.^	15.1 ± 0.7 ^n.s.^	42.6 ± 4.4 ^n.s.^
Fr D (flavone aglycones)	10	15.6 ± 1.2 ^n.s.^	15.1 ± 0.6 ^n.s.^	42.3 ± 5.0 ^n.s.^
	50	15.7 ± 1.1 ^n.s.^	15.2 ± 0.6 ^n.s.^	42.7 ± 5.5 ^n.s.^

n.s., not statistically significant (*p* > 0.05, compared with control).

**Table 5:** Comparative effects of extracts (E1–E3) and flavonoid fractions (A–D) of dandelion fruits (tested concentration-50 µg/mL) on biological properties of plasma and blood platelets.

Experiment	E1 (Total Extract)	E2 (Phenolic Acid Extract)	E3 (Flavonoid Extract)	Fr A (Luteolin)	Fr B (Philonotisflavone)	Fr C (Flavonolignans)	Fr D (Flavone Aglycones)
**Plasma**							
Lipid peroxidation induced by H_2_O_2_/Fe	Positive action (antioxidative potential)	Positive action (antioxidative potential)	Positive action (antioxidative potential)	Positive action (antioxidative potential)	No effect	No effect	Positive action (antioxidative potential)
Oxidation of protein thiols induced by H_2_O_2_/Fe	No effect	No effect	No effect	No effect	Positive action (antioxidative potential)	No effect	No effect
Protein carbonylation induced by H_2_O_2_/Fe	Positive action (antioxidative potential)	Positive action (antioxidative potential)	Positive action (antioxidative potential)	Positive action (antioxidative potential)	Positive action (antioxidative potential)	Positive action (antioxidative potential)	Positive action (antioxidative potential)
**Blood platelets**							
Lipid peroxidation in resting platelets	No effect	No effect	No effect	No effect	No effect	No effect	No effect
Lipid peroxidation in thrombin-activated platelets	No effect	No effect	Positive action (anti-platelet potential)	Positive action (anti-platelet potential)	Positive action (anti-platelet potential)	Positive action (anti-platelet potential)	Positive action (anti-platelet potential)
Lipid peroxidation in platelets treated with H_2_O_2_/Fe	Positive action (antioxidative potential)	Positive action (antioxidative potential)	Positive action (antioxidative potential)	Positive action (antioxidative potential)	Positive action (antioxidative potential)	Positive action (antioxidative potential)	Positive action (antioxidative potential)
Oxidation of protein thiols in platelets treated with H_2_O_2_/Fe	No effect	No effect	Positive action (antioxidative potential)	Positive action (antioxidative potential)	No effect	No effect	No effect
Protein carbonylation in platelets treated with H_2_O_2_/Fe	Positive action (antioxidative potential)	Positive action (antioxidative potential)	No effect	No effect	No effect	No effect	No effect
Adhesion of thrombin-activated platelets to fibrinogen	No effect	Positive action (anti-platelet potential)	Positive action (anti-platelet potential)	Positive action (anti-platelet potential)	Positive action (anti-platelet potential)	Positive action (anti-platelet potential)	No effect
Adhesion of ADP-activated platelets to fibrinogen	No effect	No effect	No effect	Positive action (anti-platelet potential)	Positive action (anti-platelet potential)	Positive action (anti-platelet potential)	No effect
GPIIb/IIIa expression–non-stimulated platelets	ND	No effect	No effect	No effect	ND	ND	ND
GPIIb/IIIa expression–platelets activated by 10 µM ADP	ND	No effect	No effect	No effect	ND	ND	ND
GPIIb/IIIa expression–platelets activated by 20 µM ADP	ND	Positive action (anti-platelet potential)	No effect	No effect	ND	ND	ND
GPIIb/IIIa expression–platelets activated by 10 µg/mL collagen	ND	Positive action (anti-platelet potential)	No effect	Positive action (anti-platelet potential)	ND	ND	ND
P-selectin expression–non-stimulated platelets	ND	No effect	No effect	No effect	ND	ND	ND
P-selectin expression–platelets activated by 10 µM ADP	ND	No effect	No effect	Positive action (anti-platelet potential)	ND	ND	ND
P-selectin expression–platelets activated by 20 µM ADP	ND	No effect	No effect	No effect	ND	ND	ND
P-selectin expression–platelets activated by 10 µg/mL collagen	ND	Positive action (anti-platelet potential)	No effect	Positive action (anti-platelet potential)	ND	ND	ND

ND, not determined.

## Supplementary Materials

The following are available online. Figure S1: Flow diagram of extraction and fractionation of dandelion fruits, Figure S2: Structures of 14 fully identified metabolites in methanol extract and prepared phenolic preparations of *Taraxacum officinale* fruits.

## Abbreviations

AA: arachidonic acid; ADP, adenosine diphosphate; APTT, activated partial thromboplastin time; BAPA, benzylsulfonyl-D-arginyl-prolyl-4-amidinobenzylamide; BSA, bovine serum albumin; CAD, charged aerosol detector; COX, cyclooxygenase; CPDA, citrate/phosphate/dextrose/adeninę; DAD, diode array detector; DMSO, dimethylsulfoxide; DNPH, 2,4-dinitrophenylhydrazine; DPPH, 2,2-diphenyl-1-picrylhydrazyl; DTNB, 5,5′-dithiobis-(2-nitrobenzoic) acid; DW, dry weight; EDTA, ethylenediaminetetraacetic acid; Fg, fibrynogen; GAE, gallic acid equivalents; H_2_O_2_, hydrogen peroxide; HCAs, hydroxycinnamic acid esters; HPLC, high-performance liquid chromatography; HR-QTOF-MS, high resolution-quadrupole time of flight-mass spectrometry; LC-MS, liquid chromatography-mass spectrometry; LDH, lactate dehydrogenase; MDA, malondialdehyde; NADH, nicotinamide-adenine-dinucleotide; PGG_2_, prostaglandin peroxide G_2_; PGH_2_, prostaglandin peroxide H_2_; PT, prothrombin time; ROS, reactive oxygen species; SPE, solid phase extraction; TBARS, thiobarbituric acid reactive substances; TE, trolox equivalents; TPC, total phenolic content; TT, thrombin time; T-TAS, total thrombus formation analysis system.

## References

[1] Gale A.J. (2011). Continuing education course #2: Current understanding of hemostasis. Toxicol. Pathol..

[2] Kluft C., Burggraaf J. (2011). Introduction to hemostasis from a pharmacodynamic perspective. Br. J. Clin. Pharm..

[3] Qiao J., Arthur J.F., Gardiner E.E., Andrews R.K., Zeng L., Xu K. (2018). Regulation of platelet activation and thrombus formation by reactive oxygen species. Redox Biol..

[4] Bartosz G. (2013). Druga Twarz Tlenu. Wyd. 2.

[5] Ivanov I.G. (2014). Polyphenols content and antioxidant activities of *Taraxacum officinale* F.H. Wigg (dandelion) leaves. Int. J. Pharm. Phytochem. Res..

[6] DRUGBANK. https://www.drugbank.ca/categories/DBCAT000368.

[7] Lis B., Olas B. (2019). Pro-health activity of dandelion (*Taraxacum officinale* L.) and its food products–history and present. J. Funct. Foods.

[8] Martinez M., Poirrier P., Chamy R., Prüfer D., Schulze-Gronover C., Jorquera L., Ruiz G. (2015). *Taraxacum officinale* and related species—An ethnopharmacological review and its potential as a commercial medicinal plant. J. Ethnopharmacol..

[9] Majewski M., Lis B., Juśkiewicz J., Ognik K., Borkowska-Sztachańska M., Jedrejek D., Stochmal A., Olas B. (2020). Phenolic fractions from dandelion leaves and petals as modulators the lipid profile and antioxidant status in an in vivo study. Antioxidants.

[10] Jedrejek D., Lis B., Rolnik A., Stochmal A., Olas B. (2019). Comparative phytochemical, cytotoxicity, antioxidant and hemostatic studies of *Taraxacum officinale* root preparations. Food Chem. Toxicol..

[11] Xue Y., Zhang S., Du M., Zhu M.J. (2017). Dandelion extract suppresses reactive oxidative species and inflammasome in intestinal epithelial cells. J. Funct. Foods.

[12] Williams C.A., Goldstone F., Greenham J. (1996). Flavonoids, cinnamic acids and coumarins from the different tissues and medicinal preparations of *Taraxacum officinale*. Phytochemistry.

[13] Sareedenchai V., Zidorn C. (2010). Flavonoids as chemosystematic markers in the tribe *Cichorieae* of the *Asteraceae*. Biochem. Syst. Ecol..

[14] Colle D., Arantes L.P., Rauber R., Campos De Mattos S.E., Teixeira Rocha J.B., Nogueira C.W., Soares F.A. (2012). Antioxidant properties of *Taraxacum officinale* fruit extract are involved in the protective effect against cellular death induced by sodium nitroprusside in brain of rats. Pharm. Biol..

[15] SIRIUS 4. https://bio.informatik.uni-jena.de/sirius/.

[16] Lis B., Jedrejek D., Moldoch J., Stochmal A., Olas B. (2019). The anti-oxidative and hemostasis-related multifunctionality of L-chicoric acid, the main component of dandelion: An in vitro study of its cellular safety, antioxidant and anti-platelet properties, and effect on coagulation. J. Funct. Foods.

[17] Lis B., Rolnik A., Jedrejek D., Soluch A., Stochmal A., Olas B. (2019). Dandelion (*Taraxacum officinale* L.) root components exhibit anti-oxidative and antiplatelet action in an in vitro study. J. Funct. Foods.

[18] Jedrejek D., Kontek B., Lis B., Stochmal A., Olas B. (2017). Evaluation of antioxidant activity of phenolic fractions from the leaves and petals of dandelion in human plasma treated with H_2_O_2_ and H_2_O_2_/Fe. Chem. Biol. Interact..

[19] Schütz K., Carle R., Schieber A. (2006). *Taraxacum*: A review on its phytochemical and pharmacological profile. J. Ethnopharmacol..

[20] Schütz K., Kammerer D.R., Carle R., Schieber A. (2005). Characterization of phenolic acids and flavonoids in dandelion (*Taraxacum officinale* WEB. ex WIGG.) root and herb by high-performance liquid chromatography/electrospray ionization mass spectrometry. Rapid Commun. Mass Spectrom..

[21] Mercader A.G., Pomilio A.B. (2012). Biflavonoids: Occurrence, Structural Features and Bioactivity.

[22] Geiger H., Bokel M. (1989). Die Biflavonoidausstattung von *Chimarrhis turbinate* (Hedw.). Z. Nat..

[23] Seeger T., Geiger H., Zinsmeister H.D. (1991). Bartramiaflavone, a macrocyclic biflavonoid from the moss *Bartramia pomiformis*. Phytochemistry.

[24] Zhang B.B., Dai Y., Liao Z.X. (2011). Chemical Constituents of *Saussurea eopygmaea*. Chin. J. Nat. Med..

[25] Lone S.H., Khuroo M.A. (2016). Biflavanoids: Chemical and Pharmacological Aspects.

[26] Choi J., Yoon K.D., Kim J. (2018). Chemical constituents from *Taraxacum officinale* and their α-glucosidase inhibitory activities. Bioorganic Med. Chem. Lett..

[27] Rice-Evans C.A., Miller N.J., Paganga G. (1996). Structure-antioxidant activity relationships of flavonoids and phenolic acids. Free Radic. Biol. Med..

[28] Karolczak K., Olas B., Kołodziejczyk J. (2009). The role of thiols in blood platelet activation. Post. Biol. Komorki.

[29] Łapaciuk S. (1996). Zakrzepy i Zatory. Wyd. 1.

[30] Windyga J. (2006). Skazy Krwotoczne. Wyd. 1.

[31] Kim K., Park K. (2019). A review of antiplatelet activity of traditional medicinal herbs on integrative medicine studies. Evid. Based Complement. Altern. Med..

[32] Kaikita K., Hosokawa K., Dahlen J.R., Tsujita K. (2019). Total thrombus-formation analysis system (T-TAS): Clinical application of quantitative analysis of thrombus formation in cardiovascular disease. Thromb. Hemost..

[33] Versteeg H. (2013). New fundamentals in hemostasis. Physiol. Rev..

[34] Benavente-Garcia O., Castillo J. (2008). Update on uses and properties of citrus flavonoids: New findings in anticancer, cardiovascular, and anti-inflammatory activity. J. Agric. Food Chem..

[35] Guerrero J.A., Navarro-Nunez L., Lozano M.L., Martinez C., Vicente V., Gibbins J.M., Rivera J. (2007). Flavonoids inhibit the platelet TxA(2) signaling pathway and antagonize TxA(2) receptors (TP) in platelets and smooth muscle cells. Br. J. Clin. Pharm..

[36] Dell’Agli M., Maschi O., Galli G.V., Fagnani R., Da Cero E., Caruso D., Bosino E. (2008). Inhibition of platelet aggregation by olive oil phenols via cAMP-phosphodiesterase. Br. J. Nutr..

[37] Manach C., Scalbert A., Morand C., Rémésy C., Jiménez L. (2004). Polyphenols: Food sources and bioavailability. Am. J. Clin. Nutr..

[38] Manach C., Williamson G., Morand C., Scalbert A., Rémésy C. (2005). Bioavailability and bioefficacy of polyphenols in humans. I. Review of 97 bioavailability studies. Am. J. Clin. Nutr..

[39] Makowska-Wąs J., Janeczko Z. (2004). Bioavailability of plant polyphenols. Post. Fitoter..

[40] Baeza G., Bachmair E.M., Wood S., Mateos R., Bravo L., De Roos B. (2017). The colonic metabolites dihydrocaffeic acid and dihydroferulic acid are more effective inhibitors of in vitro platelet activation than their phenolic precursors. Food Funct..

[41] Doolittle R.F., Schubert D., Schwartz S.A. (1967). Amino acid sequence studies on artiodactyl fibrinopeptides I Dromedary camel, mule deer, and cape buffalo. Arch. Biochem. Biophys..

[42] Brand-Williams W., Cuvelier M.E., Berset C. (1995). Use of a free radical method to evaluate antioxidant activity. Lebensm. Wiss. Technol..

[43] Walkowiak B., Michalak E., Koziołkiewicz W., Cierniewski C.S. (1989). Rapid photometric method for estimation of platelet count in blood plasma or platelet suspension. Thromb. Res..

[44] Whitaker J.R., Granum P.E. (1980). An absolute method for protein determination based on difference in absorbance at 235 and 280 nm. Anal. Biochem..

[45] Bellavite P., Andrioli G., Guzzo P., Arigliano P., Chirumbolo S., Manzato F., Santonastaso C. (1994). A colorimetric method for the measurement of platelet adhesion in microtiter plates. Anal. Biochem..

[46] Rywaniak J., Luzak B., Watała C. (2012). The advantage of flow cytometry technique for evaluation of viability of blood platelets stained with calcein. J. Lab. Diagn..

[47] Malinowska J., Kołodziejczyk-Czepas J., Moniuszko-Szajwaj B., Kowalska I., Oleszek W., Stochmal A., Olas B. (2012). Phenolic fractions from *Trifolium pallidum* and *Trifolium scabrum* aerial parts in human plasma protect against changes induced by hyperhomocysteinemia. Food Chem. Toxicol..

[48] Hosokawa K., Ohnishi T., Kondo T., Fukasawa M., Koide T., Maruyama I., Tanaka K. (2011). A novel automated microchip flow-chamber system to quantitatively evaluate thrombus formation and antithrombotic agents under blood flow conditions. J. Thromb. Hemost..

[49] Wroblewski F., Ladue J.S. (1955). Lactic dehydrogenase activity in blood. Proc. Soc. Exp. Biol. Med..

[50] Wachowicz B. (1984). Adenine nucleotides in thrombocytes of birds. Cell Biochem. Funct..

[51] Levine R.L., Garland D., Oliver C.N., Amici A., Climent I., Lenz A.-G., Ahn B.-W., Shaltiel S., Stadtman E.R. (1990). Determination of carbonyl content in oxidatively modified proteins. Methods Enzymol..

[52] Ando Y., Steiner M. (1973). Sulphydryl and disulphide groups of platelet membranes: Determination of sulphydryl groups. Biochim. Biophys. Acta.

[53] Ando Y., Steiner M. (1973). Sulphydryl and disulphide groups of platelet membranes: Determination of disulphide groups. Biochim. Biophys. Acta.

