# Supplementary material for: Flavonoid Preparations from Taraxacum officinale L. Fruits—A Phytochemical, Antioxidant and Hemostasis Studies
Source: Molecules. 2020 Nov 18;25(22):5402. doi: 10.3390/molecules25225402 (PMC7699217; doi:10.3390/molecules25225402)
Supplement: Supplementary file 1 [file molecules-25-05402-s001.pdf]

Supplementary Materials

Article

Flavonoid Preparations from *Taraxacum officinale* L. Fruits-a Phytochemical, Antioxidant and Hemostasis Studies

Bernadetta Lis [1], Dariusz Jedrejek [2], Joanna Rywaniak [3], Agata Soluch [2], Anna Stochmal [2] and Beata Olas [1,*]

[1] Department of General Biochemistry, Faculty of Biology and Environmental Protection, University of Lodz, 90-236 Lodz, Poland; bernadetta.lis@biol.uni.lodz.pl

[2] Department of Biochemistry and Crop Quality, Institute of Soil Science and Plant Cultivation, State Research Institute, 24-100 Pulawy, Poland; djedrejek@iung.pulawy.pl (D.J.); asoluch@iung.pulawy.pl (A.S.); asf@iung.pulawy.pl (A.S.)

[3] Department of Immunology and Infectious Biology, Institute of Microbiology, Biotechnology and Immunology, Faculty of Biology and Environmental Protection, University of Lodz, 90-237 Lodz, Poland; joanna.rywaniak@biol.uni.lodz.pl

* Correspondence: beata.olas@biol.uni.lodz.pl

Received: 14 October 2020; Accepted: 15 November 2020; Published: 18 November 2020

Table of Contents:

Figure S1. Flow diagram of extraction and fractionation of dandelion fruits
Figure S2. Structures of 14 fully identified metabolites in methanol extract and prepared phenolic preparations of *Taraxacum officinale* fruits

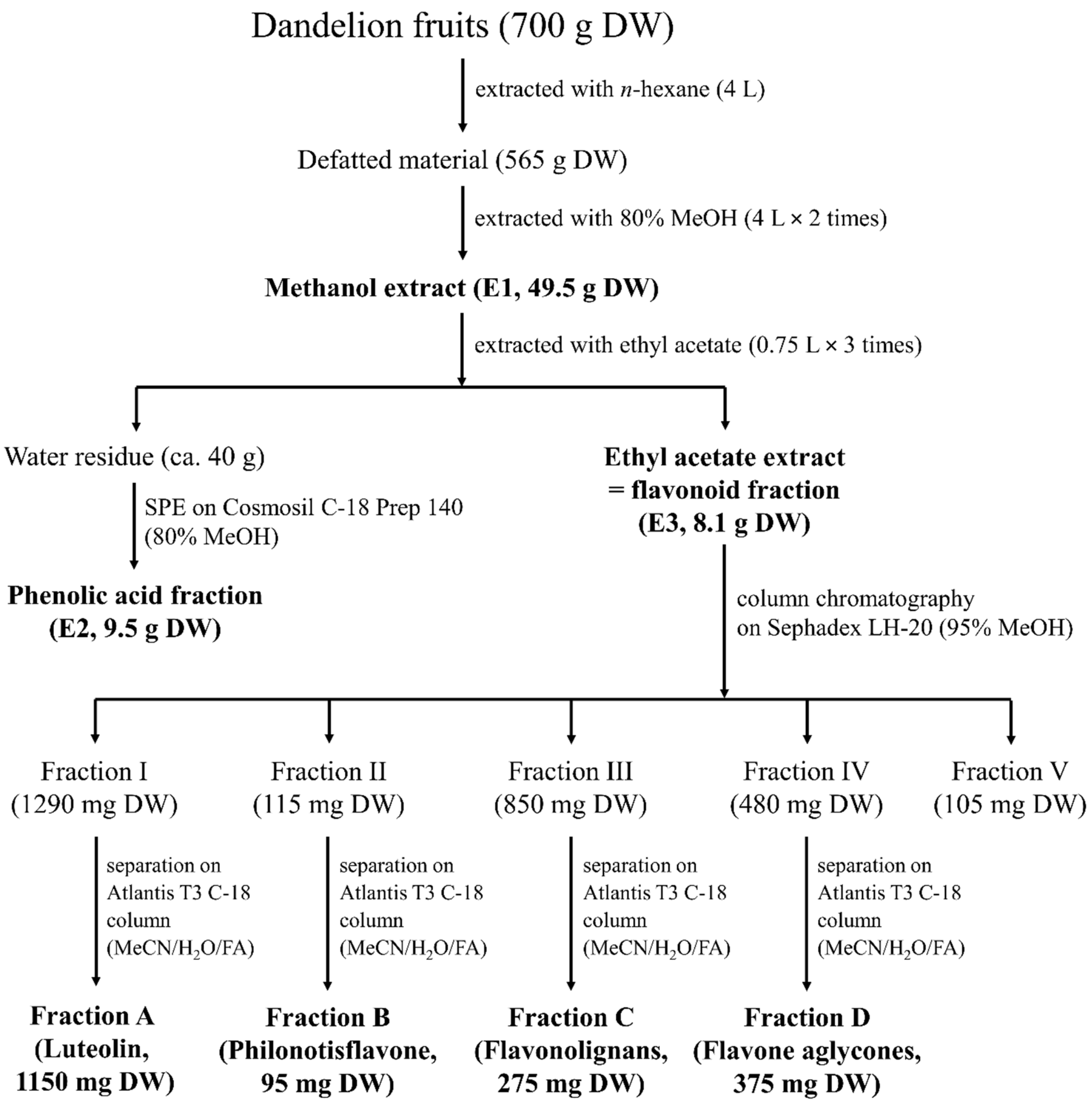

Figure S1. Flow diagram of extraction and fractionation of dandelion fruits

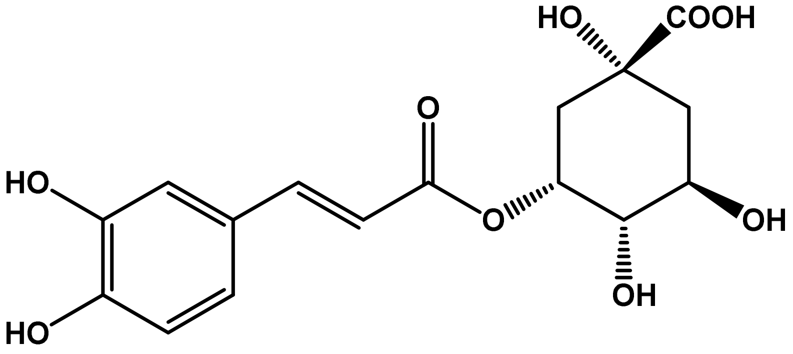

5-*O*-caffeoylquinic acid (MW = 354)

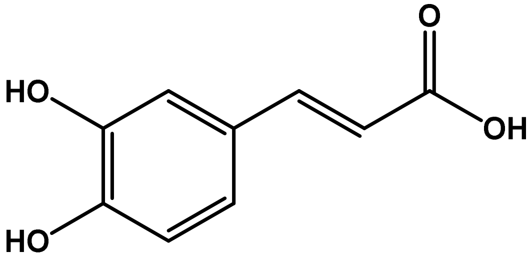

caffeic acid (MW = 180)

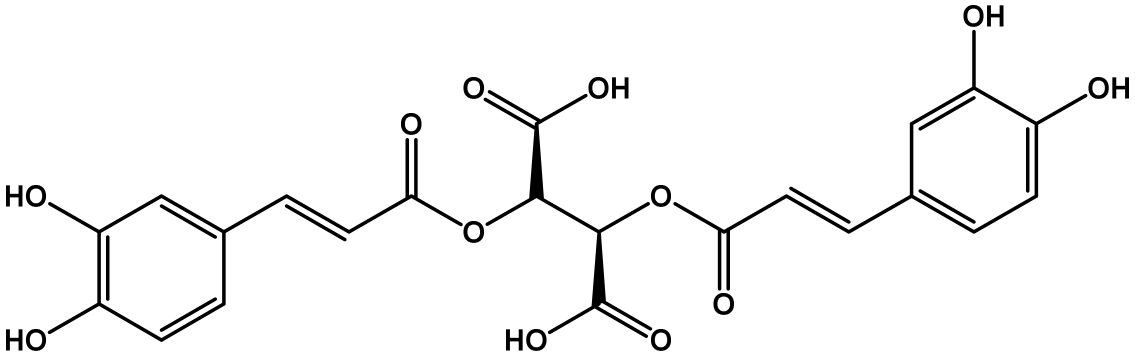

L-chicoric acid (MW = 474)

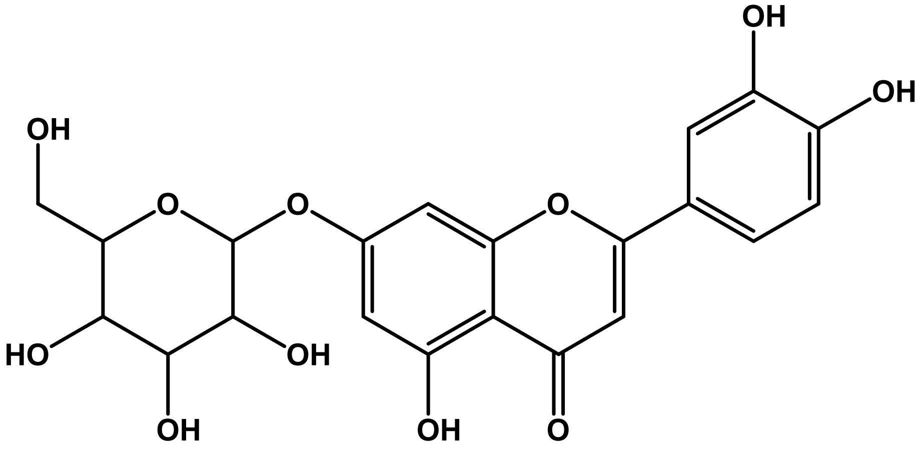

luteolin 7-*O*-glucoside (MW = 448)

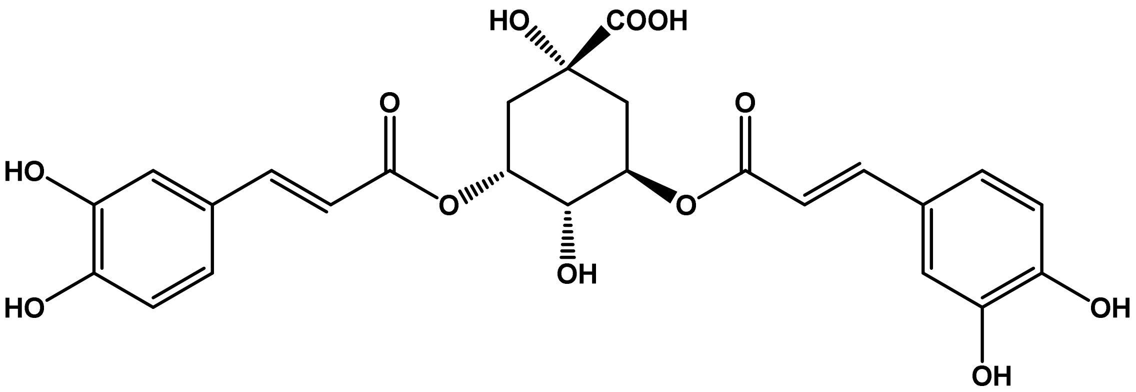

3,5-di-caffeoylquinic acid (MW = 516)

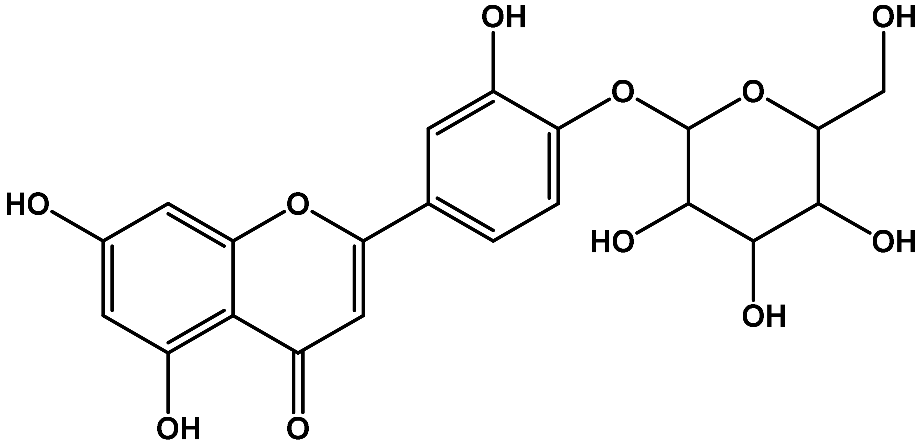

luteolin 4'-*O*-glucoside (MW = 448)

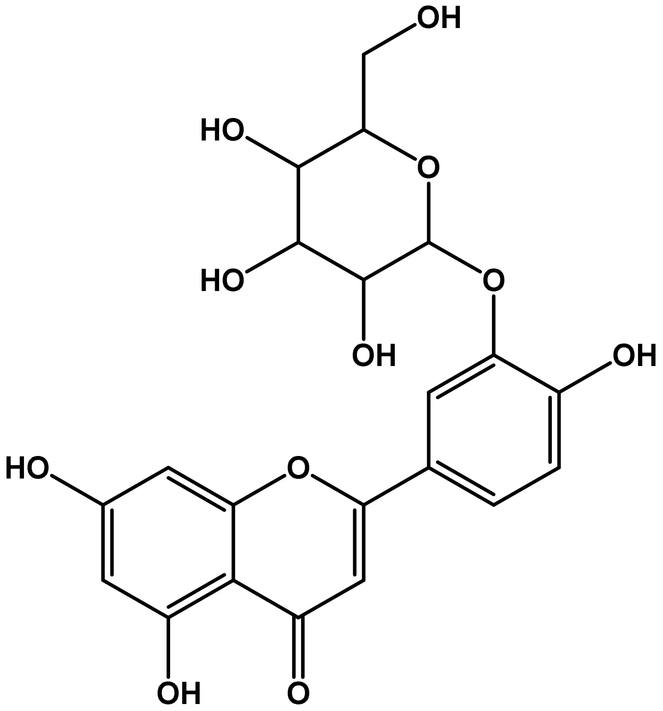

luteolin 3'-*O*-glucoside (MW = 448)

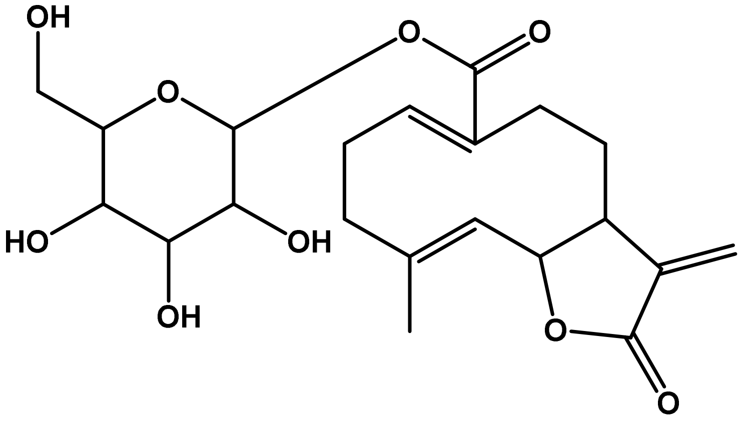

taraxinic acid 1'-*O*-glucoside (MW = 424)

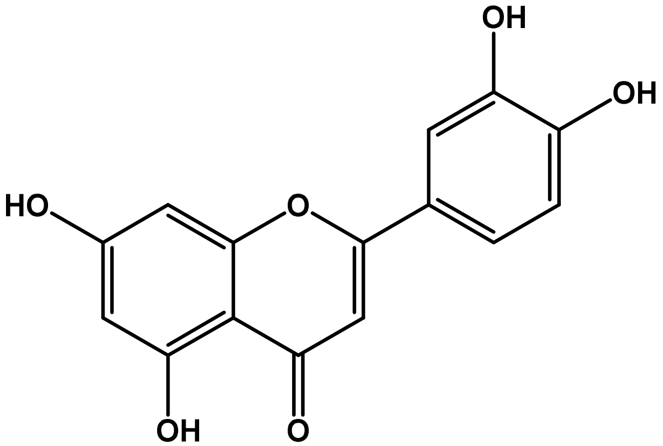

luteolin (MW = 286)

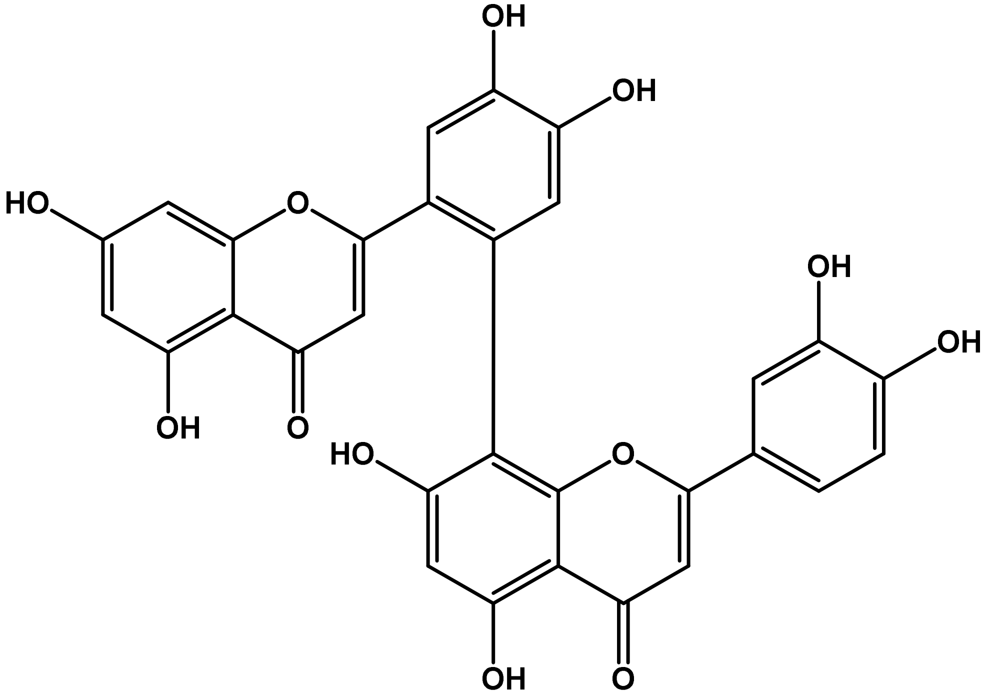

philonotisflavone (MW = 570)

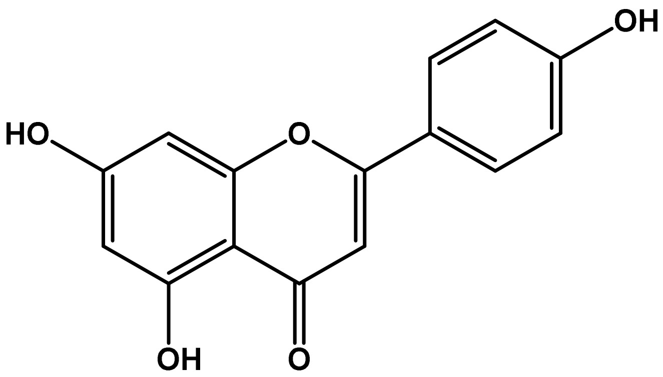

apigenin (MW = 270)

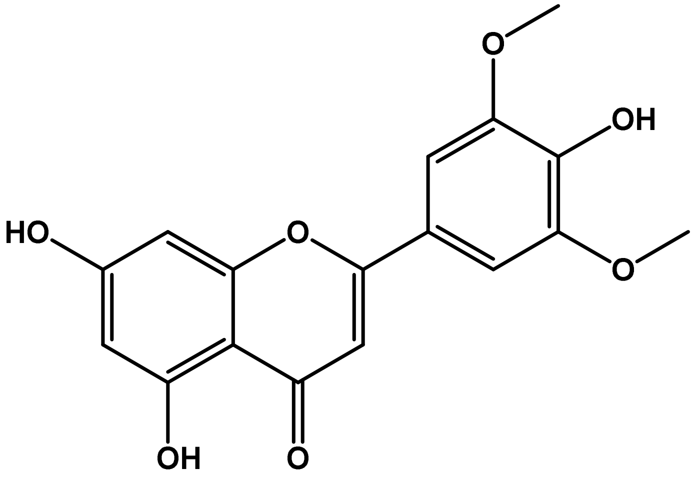

tricin (MW = 330)

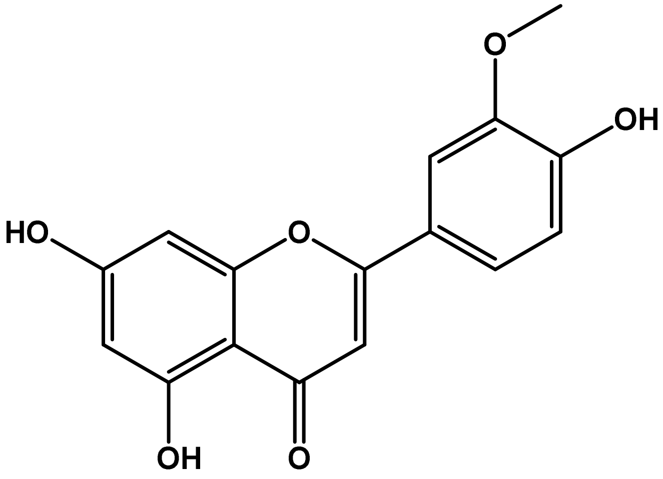

chrysoeriol (MW = 300)

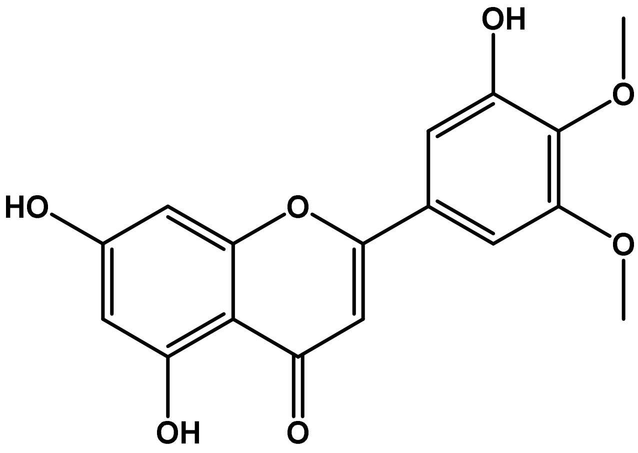

apometzgerin (MW = 330)

Figure S2. Structures of 14 fully identified metabolites in methanol extract and prepared phenolic preparations of *Taraxacum officinale* fruits
